# MCbiclust: a novel algorithm to discover large-scale functionally related gene sets from massive transcriptomics data collections

**DOI:** 10.1093/nar/gkx590

**Published:** 2017-07-14

**Authors:** Robert B. Bentham, Kevin Bryson, Gyorgy Szabadkai

**Affiliations:** 1 Department of Cell and Developmental Biology, Consortium for Mitochondrial Research, University College London, London WC1E 6BT, UK; 2 The Francis Crick Institute, London NW1 1AT, UK; 3 Department of Computer Sciences, University College London, London WC1E 6BT, UK; 4 Department of Biomedical Sciences, University of Padua, 35131 Padua, Italy

## Abstract

The potential to understand fundamental biological processes from gene expression data has grown in parallel with the recent explosion of the size of data collections. However, to exploit this potential, novel analytical methods are required, capable of discovering large co-regulated gene networks. We found current methods limited in the size of correlated gene sets they could discover within biologically heterogeneous data collections, hampering the identification of multi-gene controlled fundamental cellular processes such as energy metabolism, organelle biogenesis and stress responses. Here we describe a novel biclustering algorithm called Massively Correlated Biclustering (MCbiclust) that selects samples and genes from large datasets with maximal correlated gene expression, allowing regulation of complex networks to be examined. The method has been evaluated using synthetic data and applied to large bacterial and cancer cell datasets. We show that the large biclusters discovered, so far elusive to identification by existing techniques, are biologically relevant and thus MCbiclust has great potential in the analysis of transcriptomics data to identify large-scale unknown effects hidden within the data. The identified massive biclusters can be used to develop improved transcriptomics based diagnosis tools for diseases caused by altered gene expression, or used for further network analysis to understand genotype-phenotype correlations.

## INTRODUCTION

Gene expression datasets can now contain thousands of samples, each measuring tens of thousands of genes. Moreover, the size of the currently generated sample-gene matrices continues to increase dramatically with the advances of more economical high throughput technologies. These extensive datasets hold the promise for the discovery of novel regulatory networks underlying fundamental physiological and pathological cellular processes governed by multitudes of genes, such as cellular energy and redox metabolism, organelle biogenesis and integrated stress responses (1–5). Indeed, while quantitative models of networks involving genes on relatively small scale are now well established (e.g. see (6–9) related to metabolism), bioinformatic discovery approaches capable of handling large datasets are in critical need of development.

Currently, extracting information on biological processes from genomic, transcriptomic and proteomic datasets relies on a pipeline including (i) identification of frequent genomic mutations or differentially represented transcripts or proteins, followed by (ii) pathway and network analysis methods using gene-set, pathway or network databases (for a recent review, see (10)). A number of effective approaches for both stages of the analysis have been developed, but they have considerable limitations.

First, differential expression algorithms (11,12) are used to filter experimental data to find genes with significant alterations, producing lists that can be sorted into biologically relevant groups using gene set enrichment analyses. Recent developments, such as gProfiler (13) or GSEA (14) extended the value of this approach by considering a ranked or continuous scale of gene expression differences, as opposed to methods using unranked sets of genes chosen with fixed gene expression p-value thresholds (e.g. DAVID (15)). However, interactions and potential co-regulation of genes are not considered in these approaches, thus they can only be used to assign previously determined fixed gene sets enriched in the data. Accordingly, these methods do not allow the discovery of novel functional groups relevant to distinct physiological and pathologically states. One approach to partly overcome this limitation is to incorporate databases with rich information on gene or protein interactions, such as BioGRID (16), IntAct (17), STRING (18) or GeneMANIA (19), and identifying networks with altered gene expressions. Numerous examples using this approach exist, such as GeneMANIA (19), ReactomeFIViz (20), STRING (18), ResponseNet (21), NetBox (22), MEMo (23) and EnrichNet (24). Whilst these approaches were proven successful in identifying altered core pathways in several pathologies, they are based on prior knowledge of network components and structures, thus still have limited potential to discover novel co-regulated large-scale networks determining cellular phenotypes. In this paper we argue that large-scale differences in gene expression, for instance between different physiological and pathological states, go undiscovered due to these limitations and that novel methods discovering large-scale co-regulated gene networks are needed.

Another difficulty is that the large datasets, which these days are commonly used for network discovery, typically are not generated by experimental design based on *a priori* knowledge but are more often mass data collection projects containing vastly heterogeneous samples. In many cases it is unclear how to divide these samples into subclasses, due to the many unknown factors distinguishing subtypes with different gene expression patterns. Hierarchical clustering has notably been used to find related subgroups of samples, notably first by Eisen (25) but also by Perou (26) who used this technique to identify the intrinsic subtypes of breast cancer. These standard clustering techniques however are only useful at finding strong patterns within the data, since they cluster all the samples against all the genes or vice versa, risking to omit more global weaker patterns, due to high noise. Modes of gene regulation could be present in only a subset of samples, with genes being conditionally co-regulated only on specific cellular or environmental signals (27). With only a subset of samples having this regulation, standard clustering techniques would not detect this co-regulation in the noise of the data. Thus our second consideration for developing a method solving this problem and discriminating heterogeneous samples with co-regulated genes in large datasets was to use biclustering algorithms.

Biclustering techniques were first applied to gene expression by Cheng and Church (28), but the technique itself dates back to the 1970s in the work of Hartigan who referred to it as direct clustering (29). Biclustering algorithms select a subset of the rows and columns of a data matrix such that a particular measurement describing the quality of the bicluster is maximised. It is not known *a priori* how many significant biclusters there are within a data matrix, and the number and size of found biclusters depend on (i) the definition of bicluster (e.g. correlation of gene expression across samples), (ii) the method of measuring its quality and (iii) the method for searching for biclusters. There are a large number of existing biclustering algorithms involving different quality metrics as well as search heuristics for finding them (30), but we have found them of limited use for the scope of finding large co-regulated gene sets in a subset of samples within massive datasets. Mean square residue score for evaluating biclusters (28) is used in many biclustering techniques (MSB (31), FLOC (32), BiHEA (33) etc.). As a quality metric it does find biologically relevant biclusters but is limited to finding bicluster involving a simple shift in gene expression between samples but not patterns which involve more pronounced scaling of gene expression (34). Moreover, most of these methods are not computationally efficient on very large datasets, since finding biclusters has been shown to be an NP-hard problem (35), much more difficult than normal clustering. Accordingly, existing biclustering algorithms are adept at finding many small sized biclusters involving relatively few genes but not suitable for discovering large-scale biclusters.

Here, we describe the development of a conceptually novel biclustering algorithm, based on evaluating correlated gene expression across large sets of heterogeneous samples. The approach, in contrast to previous methods, is (i) computationally feasible to be applied to large data matrices containing whole genome transcriptomic data of more than a thousand samples, and (ii) capable to identify correlated, biologically relevant large gene sets and by including a ranking function defines subsets of heterogeneous samples where the gene set is differentially regulated. The method addresses key questions in functional genome biology. First, by quantifying correlations and expression levels of the discovered gene sets the method can be applied to classify samples. In addition, the gene sets can be used for discovery of large networks, controlled by master transcriptional regulators, which thus likely determine fundamental cellular phenotypes.

## MATERIALS AND METHODS

### The MCbiclust workflow

**Figure 1.**
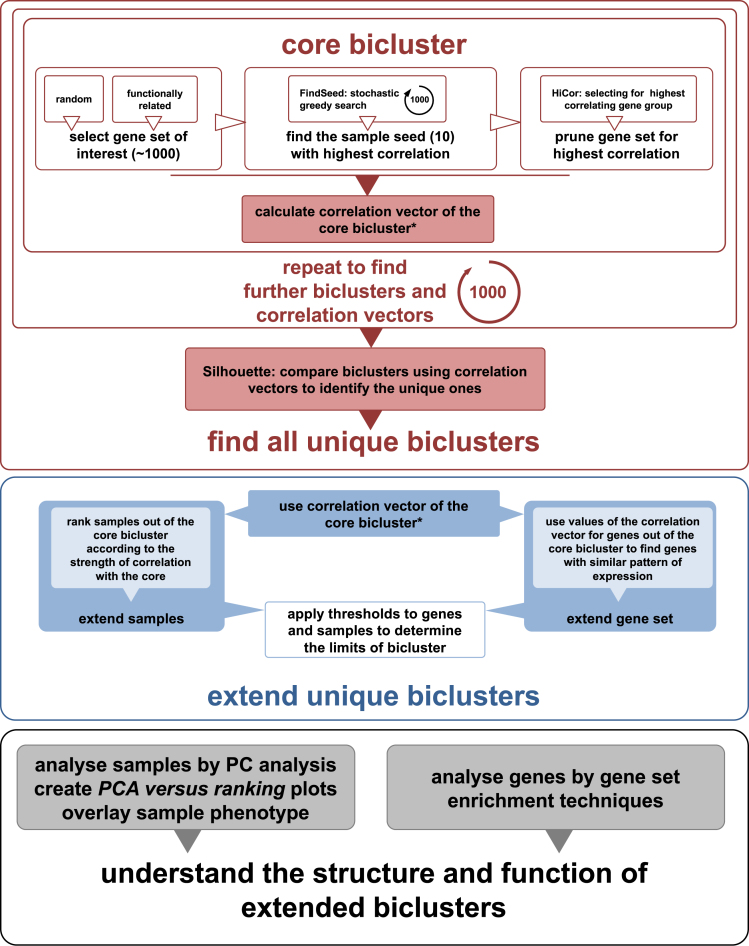
Schematic overview of the MCbiclust pipeline. The schematic shows (i) the methods used to find a core bicluster how this process is repeated and compared by Silhouette analysis to identify the unique biclusters (upper panel); (ii) how these biclusters are then extended (middle panel); and (iii) functionally and structurally analyzed (lower panel). The overall description of the process is given in the Materials and Methods section, with full details of each step describes in Supplementary Methods. A key step in the bicluster analysis is the calculation of correlation vectors, which is further explained in Supplementary Figure S1.

Massively correlated biclustering (MCbiclust) is a stochastic iterative search based method that uses Pearson's correlation coefficient as a quality metric to find biclusters (Figure 1). The input of MCbiclust is a gene expression matrix with several parameters chosen such as a gene set of interest. Different functions have been created in R to compute the different steps of the MCbiclust pipeline, the final output of MCbiclust for a given bicluster is a ranked list of samples with each sample having an associated PC1 value along with the score of each gene's relation to the bicluster in a ‘correlation vector’. Additionally a threshold of the bicluster output can be calculated to precisely define the genes and samples within the bicluster. Further functions then exist and can be applied to understand the biology of the bicluster, for instance by identifying significant gene sets associated with the bicluster.

The basic strategy of MCbiclust is to start with around 1000 seed genes and a small number of seed samples, then through random replacement of samples find a bicluster that can be then expanded. MCbiclust is specifically designed to find biclusters composed of large numbers of genes and samples within data sets. The hypothetical ideal bicluster is one whose genes are highly correlated across all samples in the bicluster, and it is not important whether these correlations are positive or negative. The algorithm is stochastic and each run will end with a different massively correlated bicluster being discovered. So generally, the method is run many times, typically up to a thousand, to discover the key large-scale biclustering structure within the given data collection. All the biclusters discovered are compared to determine how many different biclustering groupings exist.

For each individual run, the algorithm starts with an initial seed of 1000 genes that are either chosen randomly for discovering general large-scale features in the data collection, or are chosen for functional relevance to direct the discovery of biclusters (for instance a mitochondrial related gene set). Each run starts with a random seed of 10 samples. A greedy search is then undertaken where individual samples are randomly replaced by other samples, with the aim of always increasing the overall correlation score of the bicluster. Once 10 samples have been determined that maximize the bicluster correlation score, the pipeline focuses on the genes involved to further maximize this score. Hierarchical clustering of the genes is carried out, dividing the genes into eight groups with tightly correlated genes over the samples, only the genes from the group which has maximum bicluster score are kept with all the other genes being removed.

Now that the nucleus of a highly correlated bicluster has been formed, the bicluster is extended in terms of both samples and genes included. An ‘average gene expression vector’ is determined from the bicluster, by dividing the genes into groups with hierarchical clustering and finding the average gene expression of this group across the 10 samples. The correlation of every gene measured to this average gene expression vector can be calculated forming a ‘correlation vector’. The genes can then be ordered by their values in the correlation vector (see Supplementary Figure S1). Following gene extension, all the other samples within the data collection can be ranked according to how well they preserve the correlation of the bicluster. At each step, the sample that preserves most the correlation is added, until all the samples have been ranked. MCbiclust therefore returns a ranked list of the samples and genes matching the pattern found in the bicluster. In order to determine which genes and samples are in the bicluster a method to threshold the bicluster is applied as described in Supplementary Methods.

The biclusters discovered are often complex and thus we have used two key approaches to interpret them in terms of either the samples or genes involved. Samples are analyzed by doing Principal Component Analysis (PCA) across gene values across the 10 most prominent samples. The first principal component (PC1) is then used to visualize each of the samples within the bicluster ranked according to correlation. Generally such plots split the samples into two forks with anti-correlated gene expression between two groups of genes identified (see Supplementary Figure S2). The key approach employed to analyze the genes within a bicluster in order to help identify its biological nature is gene enrichment analysis. Although it can be seen later that bicluster interpretation often needs investigation driven by intuition based on considering both the samples and genes involved.

Detailed information about the algorithm can be found in Supplementary Methods and in the Vignette accompanying the Bioconductor package developed to perform custom MCbiclust analysis.

### Synthetic data and benchmarking

A preliminary synthetic dataset was created using an adapted version of the method used in (36) for the biclustering method FABIA, using the R package ‘FABIA’. This method implants a set number of multiplicative biclusters that match the FABIA model, into a dataset. This was done by creating eight separate synthetic datasets, using the FABIA model. Each dataset contained only one bicluster, on average containing approximately 500 genes and 130 samples, and each dataset was mean centered according to the genes before being combined. Eight biclusters were chosen so that the final combined synthetic dataset contained 1000 genes and 1059 samples. Enforcing sample exclusiveness to a single bicluster was done primarily to make the comparison between the different bicluster algorithms feasible. If a sample belonged to two or more biclusters, due to each bicluster affecting a large number of the genes, there would be a significant number of genes belonging to both biclusters and this overlap of genes could potentially confound the classification of samples to their correct bicluster.

MCbiclust was compared with the FABIA (36), FABIAS (36), biMax (37), CC (28), Plaid (38), ISA (39), FLOC (40), QUBIC (41), CPB (42) and CTWC (43) biclustering methods (see Supplementary Table S1) all run with default parameters. These methods were chosen due to their availability of access as R packages on Bioconductor (www.bioconductor.org), or due to similarity with MCbiclust (CPB and CTWC). CPB was run with a python script available at http://bmi.osu.edu/hpc/software/cpb/index.html and CTWC was run using software available at http://www.weizmann.ac.il/complex/compphys/software.

Following analysis of the preliminary data the three top biclustering methods (MCbiclust, ISA and FABIA) were chosen for more detailed investigation using synthetic data. This was done in two steps: (i) optimization of the parameters used in each biclustering method, (ii) application on additional synthetic datasets with the optimized parameters to investigate the effect of the number of biclusters, overlapping samples between different bicluster and noise level.

Full details of how the biclustering methods were optimized can be found in the supplementary material. The additional synthetic data sets were designed to address four properties that may affect the efficiency of the biclustering algorithm: (i) the number of biclusters in the data set, (ii) whether the biclusters have overlapping samples, (iii) the size of the bicluster and (iv) the level of noise present in the data. Full details of how these additional data sets were designed is given in the supplementary material. FABIA, ISA and MCbiclust were run on these synthetic data sets using the optimised parameters.

### Workflow to compare biclusters obtained with different methods

**Figure 2.**
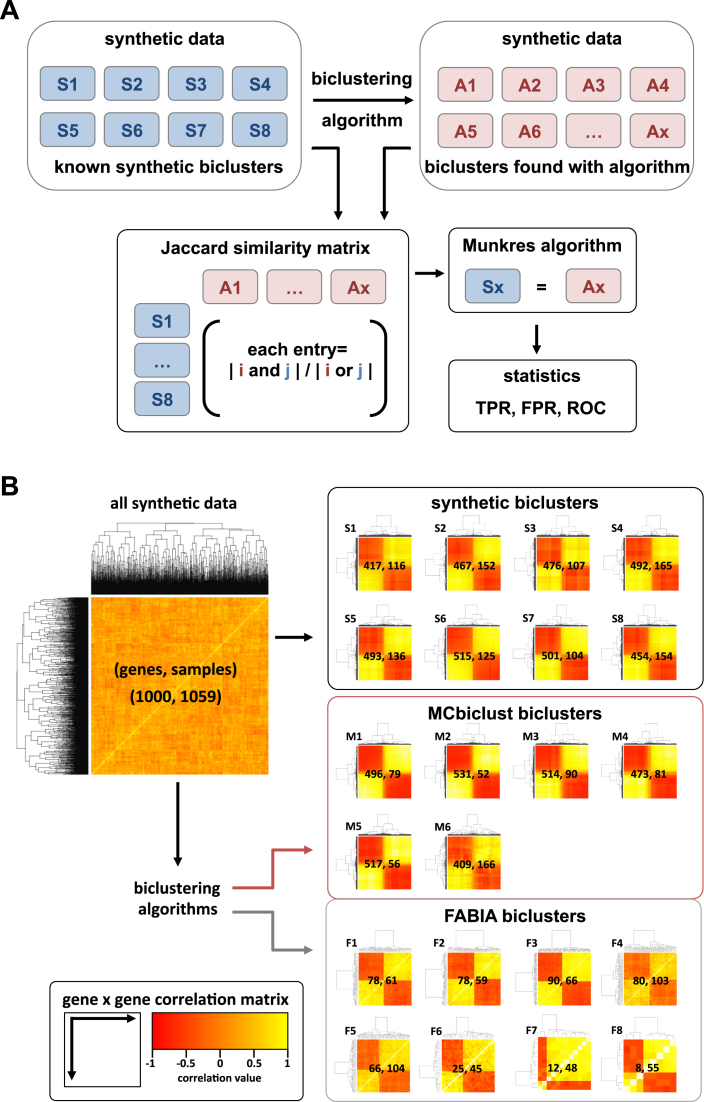
Benchmarking of MCbiclust against previous biclustering methods. (**A**) Outline of the evaluation pipeline. Known biclusters in the synthetic datasets are compared with the biclusters found with different biclustering methods. Jaccard Index and the Munkres algorithm is used to solve the assignment problem of matching the known synthetic biclusters with the found biclusters, from which statistical evaluations such as true and false positive rates (TPR, FPR) and relative operating characteristics (ROC) curves are produced. (**B**) Heatmaps of the gene-gene correlation matrices for all the synthetic data, the known synthetic biclusters (S1–S8) and the biclusters found with FABIA (F1–F8) and MCbiclust (M1–M6). Numbers of gene and samples are shown in parenthesis (gene, sample) to compare the sizes of real biclusters with the ones found with either method.

Figure 2A provides an overview of how the results of each biclustering method (shown as biclusters A1 to A*x*, where *x* is the variable number of biclusters predicted) were compared to the real biclusters present in the synthetic data (shown as S1 to S8). First, a similarity matrix is constructed where all possible predicted biclusters from the results are compared to all of the eight known biclusters in the synthetic data. The Jaccard score is used since this is appropriate for comparing the similarity between two different sets (being equal to the number of elements in the intersection of the two sets divided by the number of elements in the union of the two sets). Identical sets will have a Jaccard score of 1.0 and completely different sets will have a Jaccard score of 0.0. Once all predicted biclusters are compared to all known biclusters in the matrix, the Hungarian or Munkres algorithm (44,45) for solving the assignment problem is used to efficiently determine the most optimal matching of predicted biclusters to known biclusters which maximises the sum of the scores. At this point each real bicluster (S1 to S8) would be matched to its most optimal predicted bicluster (A1 to A8) by the method. With this matching complete, traditional measurements of accuracy, false positive and true positive scores can be used both for the samples matched and the genes matched, and receiver operating characteristic (ROC) curves can be plotted.

### Analysis on *E. coli* many microbe microarray database (M^3D^)

MCbiclust was applied to a extensive *Escherichia coli* K-12 microarray data set from the Many Microbe Microarray database (M^3D^) (46). This dataset includes 907 samples and 7459 probes measured with Affymetrix microarrays and collated from a wide range of experimental setups from 39 different researchers, uniformly normalized using robust multi-array average (RMA) (47). Faith *et al*. (46) notes that post normalisation systematic researcher biases are small relative to the biological changes present across the experimental conditions. To find biologically relevant biclusters the MCbiclust pipeline was run 1000 times on random gene sets. For additional comparison FABIA and ISA using the optimized parameters previously found were run on this dataset and compared to the MCbiclust results.

### Analysis on cancer cell line encylopedia (CCLE)

MCbiclust was applied to the CCLE dataset (48) composed of 969 samples with gene expression levels measured as mRNA using Affymetrix U133 plus 2.0 arrays and updated probe set definition files from Brainarray (49). Before analysis completed by Barretina *et al*. (48) the dataset was background corrected using RMA (47) and quantile normalization methods, with quality assessment to identify low performing microarrays. To study mitochondrial related biclusters, MCbiclust was run 1000 times on the 1098 MitoCarta (50) genes known to be related to mitochondria. MCbiclust was additionally run 1000 times on random gene sets containing 1000 genes to find biclusters affecting general pathways.

## RESULTS

### MCbiclust is uniquely designed to identify large biclusters with non-overlapping samples

In order to validate MCbiclust and compare its performance with all other selected biclustering methods using default parameters, we first used a preliminary synthetic data set, modeling large biclusters, and a custom scoring system (see Materials and Methods and Figure 2A). The dataset contained eight known biclusters (on average a matrix of 130 samples and 500 genes), and 10 biclustering methods were tested (see Supplementary Table S1). Comparison of the known biclusters with the found biclusters was carried out as previously described ((36), see Figure 2A). Based on these similarity analyses the quality of bicluster identification of each method was assessed. Table 1 shows the consensus score (36) as well as the F1 score for both genes and samples for each biclustering method as well as the number of biclusters found. The consensus score is taken from the work of Hochreiter *et al*. (36) and uses Jaccard Index similarities of the predicted biclusters to their match known biclusters, divided by the size of the larger set. In this way, the consensus score includes a penalty for finding the incorrect number of biclusters. The F1 score is the harmonic mean of the recall and precision and in general measures the accuracy of identifying the genes or samples within the bicluster.

**Table 1.** Summary statistics for comparing the different biclustering methods

Method	Biclusters Found	Consensus Score	Genes F1	Samples F1
MCbiclust optimum	6	0.4368	0.8145	0.6634
MCbiclust threshold	6	0.3462	0.8043	0.5864
FABIA	8	0.04106	0.1962	0.549
FABIAS	8	0.02475	0.2498	0.2878
biMax	8	0.002343	0.5697	0.01672
CC	8	0.0001895	0.02177	0.03344
Plaid	2	0.004164	0.1299	0.1747
ISA	504	0.001191	0.3256	0.5459
FLOC	8	0.0006008	0.06603	0.03746
QUBIC	9	0.0003819	0.008219	0.2113
CPB	24	0.0001685	0.02989	0.06277
ISA best	8	0.07504	0.3256	0.5459
CTWC	17	0.03591	0.5397	0.3329

MCbiclust optimum refers to choosing the top samples and genes that maximise the Jaccard index to the known synthetic bicluster while MCbiclust threshold is the top samples and genes chosen from MCbiclust's threshold method (see Supplementary Methods). ISA with the default parameters scans a large threshold range for bicluster size and thus a large number of biclusters, the ‘ISA best’ row indicates the result of selecting the top eight biclusters that match the known synthetic biclusters. The sizes of the known synthetic biclusters are given in Supplementary Table S3.

MCbiclust has identified six out of eight biclusters, and massively outscored the existing methods in precisely identifying large, so far hidden, biclusters within the massive dataset. This includes outperforming FABIA, whose data model was used to design the synthetic data. ISA, which is designed to be used on large datasets, found over 500 biclusters. Indeed, eight of these were reasonable matches for the synthetic bicluster, but it thus also had a very large false positive rate, by detecting small random biclusters. Even when considering only the correct eight biclusters, ISA still had a lower performance than MCbiclust. For further evaluation of the different methods, we have plotted relative operating characteristics (ROC) curves for each synthetic bicluster. These results confirmed the higher sensitivity and specificity of MCbiclust compared to methods existing so far (see Supplementary Figure S2).

**Figure 3.**
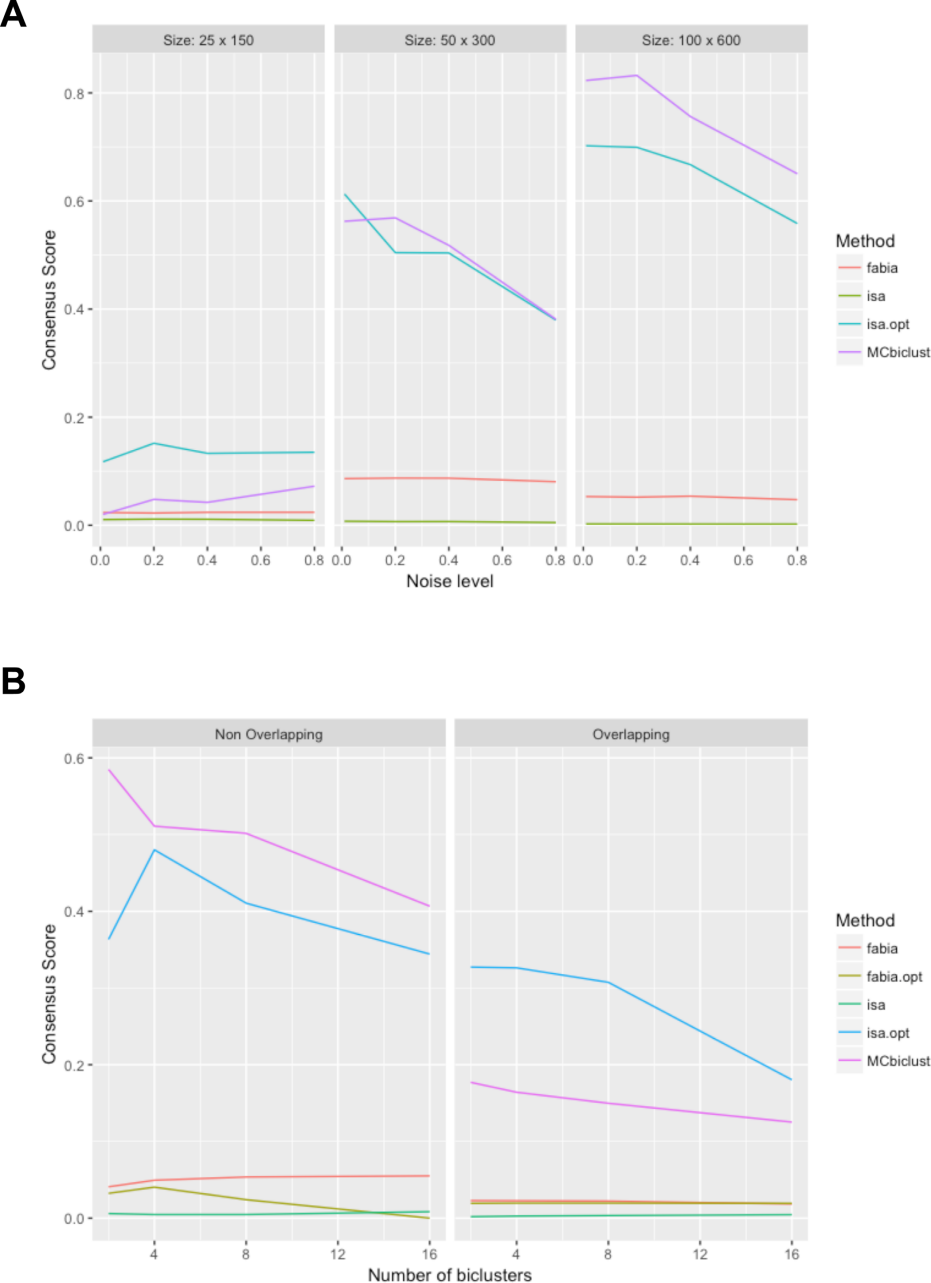
Comparison of FABIA, ISA and MCbiclust on addition synthetic data. (**A**) The effect of different sizes and levels of noise on the consensus score for the different biclustering methods including the difference between ISA with optimum and default parameters. (**B**) The effect of different number of biclusters in the consensus score in the data set, either with overlapping or non-overlapping samples. For details of the approach see Materials and Methods and Supplementary information.

Next, the top performing biclustering algorithms: MCbiclust, ISA and FABIA had their parameters optimised on a synthetic dataset (see Supplementary Figures S3 and S4), and a detailed analysis of these algorithms with optimised parameters was performed on additional synthetic data sets to investigate the effect of bicluster size, noise, number and the presence of different biclusters having overlapping samples. The results of this analysis with the consensus score used as a comparative measure is given in Figure 3. First, we determined the effect of noise and bicluster size on MCbiclust, ISA and FABIA (Figure 3A). Each individual dataset in this analysis only contained a single bicluster and was made up of 1000 genes and 1000 samples. For large biclusters containing approximately 100 samples and 600 genes MCbiclust outperformed ISA and FABIA, for biclusters containing 50 samples and 300 genes MCbiclust and ISA are comparable, and for biclusters containing 25 samples and 150 genes, ISA outperformed MCbiclust. In each case the effect of noise decreased performance. Next, we analyzed the effect of number of biclusters in the dataset and the presence of overlapping samples (Figure 3B). When the biclusters had non-overlapping samples MCbiclust outperformed both ISA and FABIA, however when the biclusters had overlapping samples, ISA was the most efficient. The decreased performance in MCbiclust is likely due to MCbiclust finding too many biclusters and identifying samples present in two biclusters as being different from samples only present in one or the other. This is expected since the correlations between samples change dramatically if they were part of more than one bicluster. Since biologically MCbiclust is concerned with gene expression programs in the cell affecting large number of genes at one time, if two or more of these programs are activated at the same time the interaction of these programs would cause significant changes in the overlapping gene set and would likely appear as a gene expression program distinct from its component biclusters.

**Figure 4.**
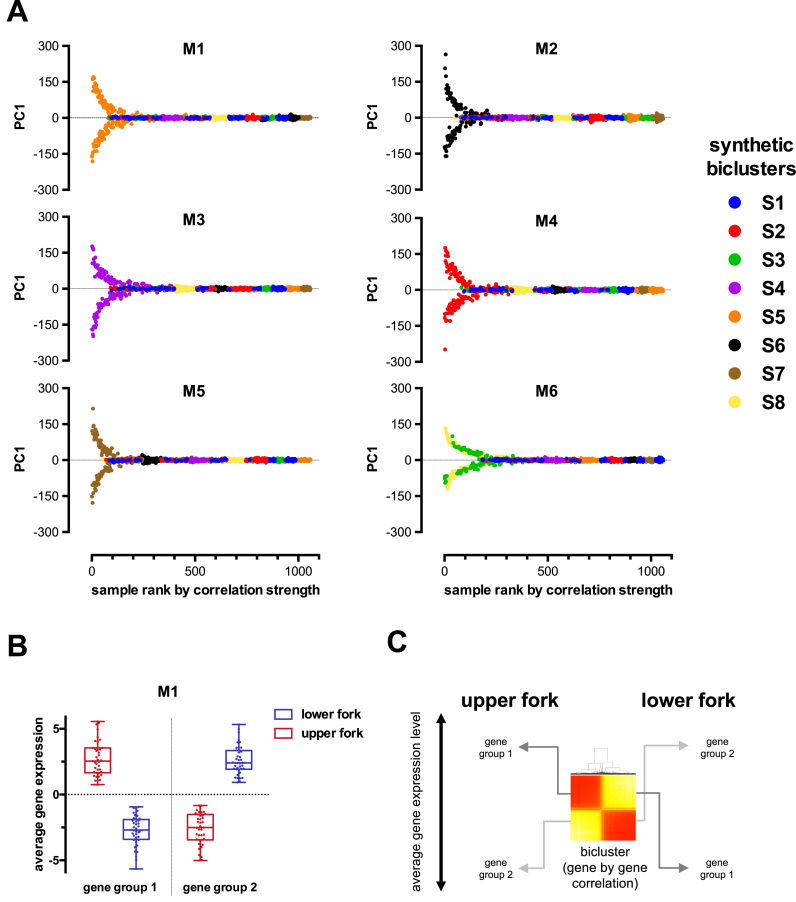
First principal component versus correlation based ranking plots of samples in biclusters identified by MCbiclust. (**A**) Fork patterns of the six biclusters found with MCbiclust in the synthetic data. Y axes show the first principal component (PC1) value for each sample in each bicluster. Principal component analysis was run on the most highly correlating samples and captures the correlation pattern present in the samples. X axes show the ordering according to how well the samples preserve this correlation. Ranking is obtained as described in the ‘Extending the bicluster – samples’ section of Supplementary Methods. (**B**) Mean centered average gene expression values of the two separate gene groups in the samples of the two forks of bicluster M1 determining the correlation. Expression levels in the two gene groups follow an antiparallel pattern. Relationship of average gene expression to PC1 values are shown in Supplementary Figure S5. (**C**) Schematics showing the gene-gene heatmaps of the M1 bicluster showing the division of the genes into two groups with different regulation in the upper and lower fork samples.

Most importantly however, MCbiclust has an additional unique feature compared to existing methods. Apart from finding biclusters, it also ranks samples according to the strength of correlation between genes found in the bicluster. Principal component analysis can thus be further used to determine subclasses of samples in the ranking space. *PCA value versus ranking* plots revealed the distribution of the clustered samples in a characteristic fork pattern (Figure 4A), probably indicating the polar distribution of samples along the average expression of the gene sets, responsible for the high correlation (see Figure 4B, C, Supplementary Figure S5 and Supplementary Methods).

### MCbiclust discovers biologically relevant gene expression patterns in *E. coli* data sets

Next, we applied the algorithm to increasingly complex gene expression datasets from heterogeneous sample collections. First, we used an extensive *E. coli* K-12 microarray data set from the Many Microbe Microarray database (M^3D^) (46). The probes of this dataset cover ORFs or transcripts of unknown function as well as non-coding intergenic regions such as operon elements, 5′-UTRs, 3′-UTRs and small RNAs. The *E. coli* K-12 model is currently the best characterised prokaryotic model for studying gene regulatory networks on different scales, including large gene sets controlled by σ factors and smaller sets by transcriptional regulators. In addition, the dataset contains a large number of annotated experimental conditions, thus it was ideal for the initial characterization of MCbiclust's ability to discover co-regulated gene sets in heterogeneous experimental conditions.

**Figure 5.**
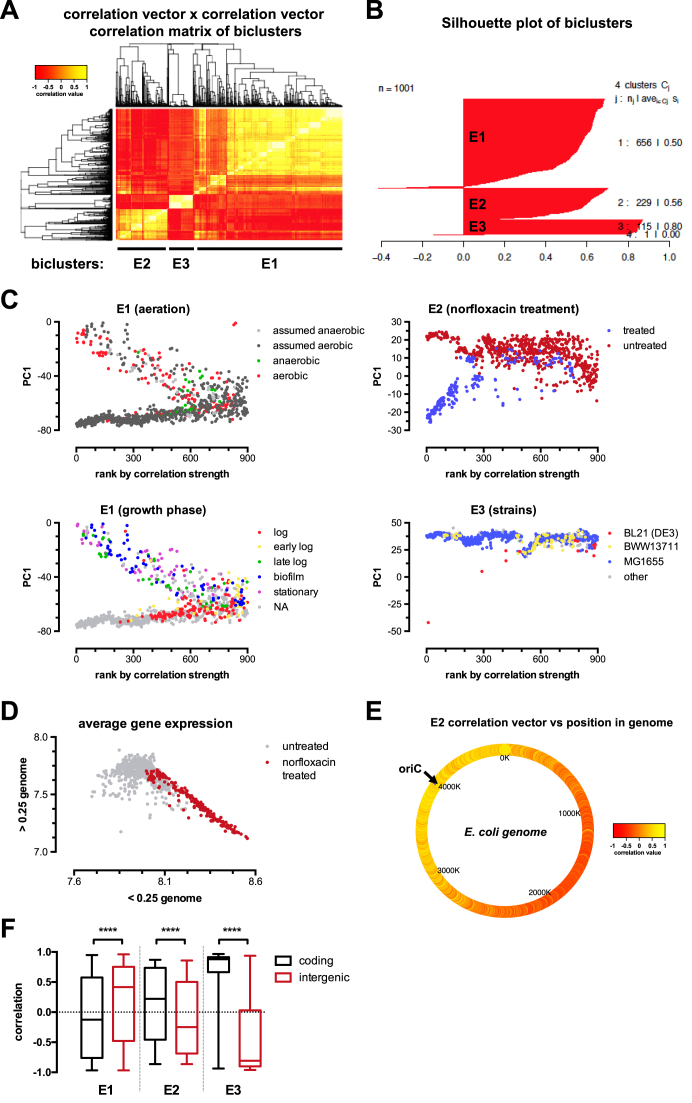
Biologically relevant biclusters discovered by MCBiclust in *E. coli*. (**A**) MCbiclust was run 1000 times on the *E. coli* K-12 microarray data set from the Many Microbe Microarray database (M^3D^). Results are visualised in a heatmap of the correlation matrix from the correlation vectors. Hierarchical clustering reveals three large bicluster groups (E1–E3). (**B**) Correlation vectors are divided into three unique bicluster groups (E1–E3) from the output of the silhouette analysis. The silhouette plot of the optimum number of clusters is shown as chosen by maximizing the average silhouette width of all the correlation vectors. (**C**) PC1 versus sample ranking plots of the unique biclusters E1, E2 and E3. The plots have been overlaid with experimental conditions: aeration and growth phases for E1 (left panels), the gyrase inhibitor norfloxacin treatment for E2 (upper right panel) and the different strains used in the experiments for E3 (lower right panel). (**D**) Plot of average gene expression values (median centered log_2_) close (<0.25 genome) versus far (>0.25 genome) to the origin of replication. The distribution of norfloxacin treated (red) and control (non treated, gray) samples are shown. (**E**) Heatmap of correlation vector values for E2 in relation to genome position (oriC, origin of replication). (**F**) Box plot of correlation vector values for all biclusters in coding (black) and intergenic (red) regions. The non-parametric Mann-Whitney test was used to calculate significance between pairs of each bicluster. ****P <0.0001

By running MCbiclust 1000 times, starting from random gene sets of 1000 genes, silhouette width analysis (51) revealed three large distinct biclusters from the resulting correlation vectors (Figure 5A and B). These groups were denoted E1, E2 and E3 and were obtained after 656, 229 and 115 runs, respectively, with the numbers indicating the runs required to reach dominance of the bicluster. These biclusters were all large; after thresholding with a sample *P*-value of 0.05 they contained 4822, 4700 and 6086 probes from 131, 130 and 96 samples, respectively.

To understand the biological relevance of these biclusters, we first analyzed the distribution of the samples in the found biclusters by PCA analysis and ranking according to the strength of correlation of gene expression (Figure 5C). As described above, the *PCA versus ranking* distribution plot typically gives a fork pattern, where the samples with highly correlated gene expressions are divided into high and low PC1 groups, where PC1 is mainly determined by the average expression level of the gene set defining the bicluster (see Supplementary Figure S5). The plot allows the classification of the samples and helps to further determine correlations with sample types and experimental conditions. As shown in Figure 5C, the samples identified in the E1 cluster were distributed along experimental conditions such as growth phase, aerobic/anaerobic status or treatment with antibiotics affecting growth. Cluster E2 clearly identified samples treated with a specific antibiotic, norfloxacin. In contrast, cluster E3 was determined by the highly deviant PC1 value associated with an outlier sample forming the lower fork of the distribution, while most of the samples remained in the upper half. Overall, the distribution analysis demonstrated the value of MCbiclust to identify biological (E1), pharmacological (E2) conditions, and outliers which otherwise would remain undetected (E3).

To identify more details of gene regulation in the biclusters we performed custom gene set enrichment analysis based on a Mann-Whitney test (see Supplementary Methods) to identify gene ontology (GO) terms related to *E. coli*, including Sigma factors and other *E. coli* transcription regulators from EcoCyc (52) and RegulonDB (53) databases. Additionally, terms for probes targeting either coding genes or intergenic regions were added. E1 and E3 had a large number of associated significant terms, 175 and 196, while E2 only had 25. Full tables of these terms are given in Supplementary Data. The custom analysis allowed the association of terms with positive and negative correlation vectors, informing on the average gene expression of pathways determining the distribution of samples in the upper or lower fork. The analysis revealed three important regulatory features.

First, the upper fork of E1 was driven by the correlated overexpression of genes with positive correlation vector values. Accordingly, those genes are predicted to drive an aerobic metabolic phenotype characteristic of slow growth in late log or stationary bacterial cultures or biofilms (see Figure 5C). The terms cover wide range of metabolic pathways comprising biosynthetic routes of all major cellular components, lipids, proteins and ribonucleotide acids (see Supplementary Data), likely representing a specific global metabolic phenotype associated with the aerobic conditions in these experiments.

Second, the significant terms from E2 are relatively few and had relatively large *P*-values. Thus we looked at additional features of the genes determining the bicluster. Intriguingly, the average correlation vector values were distributed according to the position of genes in the *E. coli* genome (Figure 5D). Indeed, Figure 5E shows that this association can be explained by up-regulation of genes close to the origin of replication, which gradually decreased with the distance from the ORI. Examination of the conditions of the samples in this bicluster (see Figure 5C) revealed that they have been grown in the presence of norfloxacin, a DNA gyrase inhibitor that prevents the division of the strands of *E. coli* DNA during replication, thus there would be two strands of DNA close to the ORI and a single strand further away, hence the gene dosage would be double around the ORI compared to genes further away resulting in this large-scale transcriptional difference in gene expression. Interestingly, a similar effect has been recently shown to exist in Streptococcus pneumonia and *E. coli* by (54).

Finally, when we examined the terms which drive correlations in all three biclusters, the most significant associations were found with probes targeting either gene encoding or intergenic regions, which showed strong anti-correlation (Figure 5F, Supplementary Data). Since average gene expression levels primarily determine PC1, our results show that expression of RNAs from intergenomic regions tend to exert inhibitory effects. This result is indicative of small non-coding regulatory RNAs that are intergenic inhibiting coding genes involved in biosynthetic processes and cell proliferation.

Altogether, MCbiclust therefore revealed three large-scale biologically relevant biclusters in the examined *E. coli* dataset: (i) one with terms linked to global metabolic changes during cellular growth in aerobic conditions, (ii) one showing how DNA gyrase targeting drug treatment stalls large-scale DNA replication and affects global gene expression and (iii) one that discovers a hidden sample preparation anomaly that seriously affects global gene expression in a single Affymetrix chip and possible other chips less severely (suggesting these chips should be removed before further analysis of this data collection). The results clearly indicate the value of MCbiclust to expose global trends in co-regulation of bacterial gene expression and other effects that cause changes in large-scale correlated gene expression within subsets of the biological samples.

Additionally, FABIA and ISA were applied to the *E. coli* data for comparison to the MCbiclust results. Neither FABIA nor ISA succeeded in identifying the same biclusters as MCbiclust, full details are given in the Supplementary Information Table S2.

### MCbiclust reveals cancer subtypes in the cancer cell line encylopedia data set

Next, in order to validate MCbiclust on highly complex and heterogeneous eukaryotic gene expression data, we have used a recently created cancer microarray dataset comprising ∼1k cancer cell lines from diverse tissues of origin (CCLE, (48)). Gene expression level heterogeneity between samples in this set arises from two main sources: (i) de-regulated gene expression triggered by the oncogenic genetic lesions and (ii) expression patterns distinctive of the tissue of origin of specific tumors. Here, due to the larger genome and sample numbers as compared to the *E. coli* dataset, we assumed that selection of the initial gene set might have substantial impact on the biclusters found and thus we have followed two different strategies. First, as described above we have run MCbiclust 1000 times utilizing random gene sets, in order to discover potential large-scale regulations affecting a subset of samples. In addition, however, we also sought to characterize specifically the regulation of multi-gene controlled global processes such as cellular metabolism and organelle biogenesis. Cancer evolution is known to involve radical rearrangements of cellular metabolism, in recent years deregulation of cellular energetics has even been recognized as an important hallmark of cancer (55). The aerobic glycolytic phenotype of many cancers for producing ATP has long been recognized, but it is less well understood how changes in mitochondrial biogenesis (here defined as co-regulation of the transcription of nuclear encoded mitochondrial genes, NEMGs) and hence energetic function affects cancer growth and survival. Thus our aim here was to investigate mitochondrial involvement in cancer using MCbiclust. Therefore, in the second instance MCbiclust was run on the CCLE dataset another 1000 times using a gene set composed of 1098 MitoCarta (50) genes, classified as NEMGs.

**Figure 6.**
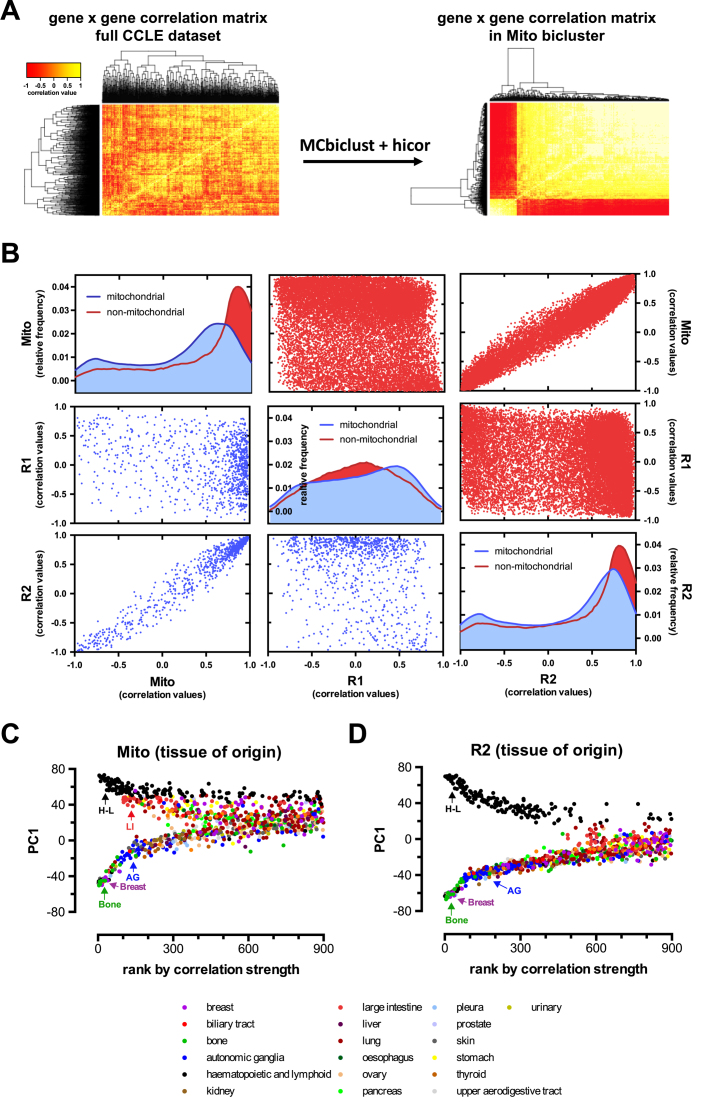
Biologically relevant biclusters in the cancer cell line encyclopedia (CCLE) microarray dataset. (**A**) Heatmaps of the MitoCarta gene–gene correlation matrices across all the samples (left panel) and in the Mito bicluster of samples and genes established by the MCbiclust and Hicor algorithms (right panel), illustrating the biclustering process (see also Figure 1 and Materials and Methods). Heatmaps and Silhouette plots of the distinct R1 and R2 biclusters identified using random initial gene sets are shown in Supplementary Figure S6. (**B**) A matrix of plots comparing the correlation vectors in all three distinct biclusters (Mito, R1 and R2). The diagonal plots show density histograms of the correlation values in the mitochondrial (blue) and non-mitochondrial gene sets (red) to the respective biclusters (Mito, upper left; R1 central; R2, lower right). Off-diagonal scatter plots show the relationships between the correlations of genes to the respective biclusters (Mito, R1 and R2, labeled left versus bottom) for mitochondrial (lower left triangle, blue) or non-mitochondrial genes (upper right triangle, red). (**C**) PC1 versus sample ranking plots of the Mito and R2 biclusters, which are highly correlated (see scatter plots in panel B). The tissue of origin of the different sample cell lines is overlaid on the distribution plot. Clustered samples with the same tissue of origin are marked in the upper (Mito: H–L: hematopoietic and lymphoid, LI: large intestine; R2: H–L: hematopoietic and lymphoid) and lower (both Mito and R2: AG: autonomic ganglia, breast, bone) forks.

Silhouette analysis identified two distinct biclusters (R1 and R2) using random gene sets and one distinct bicluster (denoted M1) when using the MitoCarta gene set (see Figure 6A and Supplementary Figure S6). These biclusters can be directly compared by plotting the average correlation vectors of each measured gene in the genome between individual biclusters, as shown in Figure 6B. Overall, we have found that the M1 and R2 biclusters are highly similar, with both having mitochondrial genes with high correlation values, thus both random and function-specific initial gene selection led to the identification of essentially the same bicluster.

**Figure 7.**
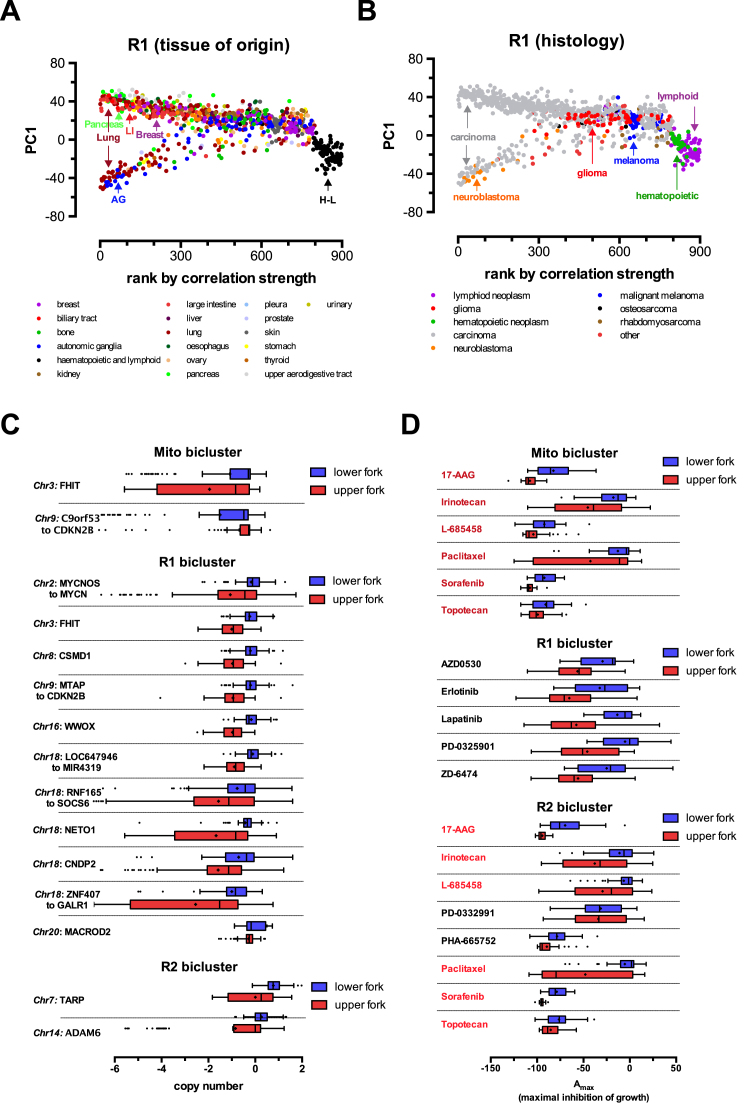
Pathological relevance of biclusters in the CCLE dataset. (A and B) PC1 versus sample ranking plots of the R1 bicluster. The tissue of origin (**A**) and tumor histology (**B**) of the different sample cell lines is overlaid on the distribution plots. Clustered samples with the same tissue of origin or histology are marked across the distribution plots (LI: large intestine, AG: autonomic ganglia, H–L: hematopoietic and lymphoid origins). (**C**) Association of copy-number differences across the whole genome with the distribution of samples in the upper and lower forks in all biclusters. Chromosome numbers and genes (labelled at left) with differences significant with a *P*-value < 0.05 are shown. (**D**) Association of differences in pharmacological sensitivity to anticancer drugs with the distribution of samples in the upper and lower forks in all biclusters. To represent pharmacological sensitivity the *A*_max_ value was used from the CCLE dataset, signifying maximum inhibition of growth for each drug treatment. Drugs (out of 24 tested, see main text and ref) with significant differences between the lower and upper forks of each bicluster are shown. All differences are significant with a *P*-value <0.05. Significance in C and D was calculated using a permutation method randomly reassigning samples to the upper and lower fork and recalculating the average difference in copy-number or *A*_max_ values between the forks, and using this to form the distribution from which the p-values were calculated.

Next we performed the same custom gene set enrichment analysis (see Materials and Methods) done on each of the average correlation vectors as with the *E. coli* data. As shown in Supplementary data, the M1 and R2 biclusters define a functional group of genes highly related to the mitochondrial respiratory chain, but also ribosomes, ribosome biogenesis. This most likely represents activation of a novel gene regulatory pathway in a subset of samples (Figure 6C and D), coupling increased mitochondrial biogenesis to cell growth. On the other hand, the R1 bicluster is highly enriched in immune system components and their regulated genes, and particularly overexpressed in a subset of carcinomas of different tissue origin (see Figure 7A and B).

Finally, we further analyzed the data to understand the potential association of the clustered gene expression patterns with the actual tissue of origin, pathology, genotype and pharmacological phenotype of the individual cancer cell lines. First, we mapped the relationship of the gene expression patterns of different cancer cell lines compared to the various biclusters. We ranked all samples according to the strength of correlations found in each bicluster, and plotted the rankings against the PC1 value for each sample. As shown above, PC1 values are mostly determined by the average gene expression values of a subgroup of genes in the bicluster (see Supplementary Figure S2). Each bicluster was thus represented by the typical fork like distribution pattern (see Figures 6C, D and 7A, B). This allowed us to overlay the tissue of origin and pathological subtype information on the distribution patterns. While the mitochondrial M1 and R2 biclusters mainly separated cancer cell lines of hematopoietic origin from the rest of the tissues, the R1 bicluster had no tissue specificity. However, this bicluster was enriched in immune system related pathways and was typical to a subset of carcinomas (see Figure 7A and B). Next, we calculated enrichment of locuses with gene copy number alterations (Figure 7C) and pharmacological sensitivity to 24 anticancer drugs utilized in the CCLE study (48) (Figure 7D). Importantly, various copy number alterations were found to be specifically associated with each bicluster, probably indicating the genetic, oncogenic origin of the gene expression patterns. Strikingly, the distribution between the upper and lower fork of the pattern also determined significant differences between the sensitivity to the growth inhibiting effects of various anticancer drugs in each bicluster (Figure 7D), indicating the potential therapeutical predicting value of MCbiclust based cancer sample classification.

### Predicting metabolic flux rearrangements based on correlating metabolic gene expression profiles by MCbiclust in human tumor samples

In our last set of analysis, we tested the capacity of MCbiclust to identify gene expression patterns of large gene sets with correlated biological function. Cellular metabolic fluxes have been shown to be partly controlled by the correlated expression of metabolic enzymes determining the specific activity of metabolic pathways (56). This control mechanism is particularly evident when metabolic pathways show remarkable plasticity to rearrange in response to defects in particular enzymes. To test whether such rearrangement can be detected at the gene expression level by MCbiclust we analyzed a dataset compiled from cancers where tumorigenesis and cancer cell metabolism is determined by the deficiency of the mitochondrial succinate dehydrogenase (SDH) enzyme. Germline mutations in the four genes encoding SDH subunits (SDHA, SDHB, SDHC and SDHD) are linked to the development of neuroendocrine tumors such as pheochromocytomas and paragangliomas. Importantly, tumors with identical pathology can also be related to VHL, RET1 and NF1 mutations, indicating a similar pathogenesis for tumor formation. However, apart from similar pathology of these cancers, a specific feature of SDH deficient tumors is the enforced rearrangement of their mitochondrial metabolism to adapt to a truncated tricarboxylic acid (TCA) cycle, which adaptation is indispensable for their growth (57).

**Figure 8.**
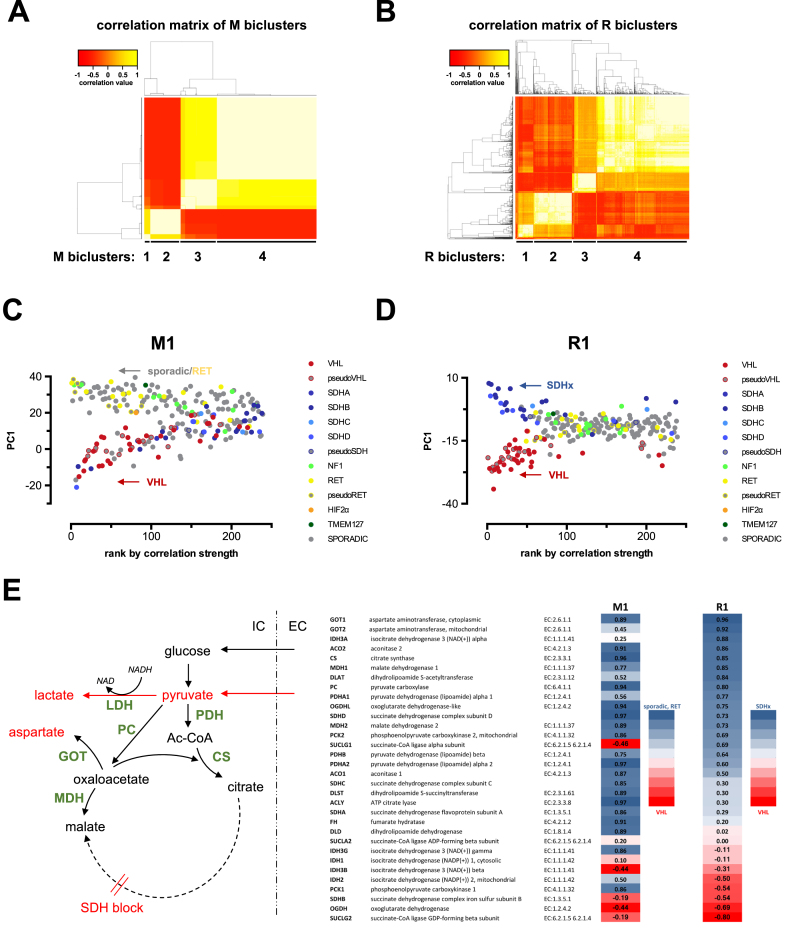
The effect of underlying germline mutations on gene expression patterns in pheochromocytoma and paraganglioma samples determined by MCbiclust. Heatmaps of the correlation matrices from the correlation vectors of the biclusters found by MCbiclust in the set of 239 pheochromocytoma and paraganglioma tumor samples collected by the Cartes d’Identité des Tumeurs (CIT) project (58) and the Erasmus MC University Medical Center Rotterdam (GSE67066), using the MitoCarta gene set (M biclusters, **A**) and random gene sets (R biclusters, **B**) are shown. Silhouette plots of the distinct M and R biclusters are shown in Supplementary Figure S7. (**C** and **D**) PC1 versus sample ranking plots of the distinct M1 and R1 biclusters. The underlying germline mutations (or sporadic - no identified mutation) is overlaid on the distribution plot. Samples clustered in the upper and lower forks of the distributions are labelled: sporadic/RET versus VHL in the M1 bicluster, and SDHx versus VHL in the R1 bicluster. (**E**) Effect of rearrangement of gene expression in the TCA cycle identified by the M1 and R1 biclusters. Scheme (left panel) shows the experimentally identified rearrangement of metabolic fluxes by Cardaci *et al.* (57). Right panel: Correlation of the expression of TCA cycle enzymes with M1 and R1 biclusters, calculated by the method described in the ‘Extending the biclusters’ section in Materials and Methods and supplementary material. Heatmap indicates the correlation of genes with the M1 bicluster, distinguishing between the forks sporadic/RET versus VHL and the R1 bicluster, distinguishing between the forks SDHx versus VHL.

In order to assess whether MCbiclust can identify gene expression patterns in a set of genes underlying mitochondrial metabolism, we have analyzed gene expression data (Affymetrix HG-U133 Plus 2.0) from a set of 239 pheochromocytoma and paraganglioma tumor samples collected by the Cartes d’Identité des Tumeurs (CIT) project (58) and the Erasmus MC University Medical Center Rotterdam (GSE67066), including 110 tumors with germline mutations in known causative genes (Figure 8). For generating the biclusters, we have followed the strategy applied to the CCLE dataset (see previous section), thus either used the MitoCarta (50) gene set of 1098 nuclear encoded mitochondrial genes, or started from thousand randomly selected gene sets. Both approaches identified multiple biclusters, Silhouette analysis identified four distinct biclusters both using the MitoCarta gene set (M1 to M4) and random gene sets (R1 to R4; Figure 8A, B and Supplementary Figure S7). By calculating the PC1 of the gene expression patterns, which is dominated by the average gene expression of the biclustered genes, and plotting it against the ranking of the sample according to the correlation strength of the genes in the biclusters in individual samples, we have generated typical fork like distribution patterns for each bicluster, and determined the distribution of the different mutations. We have found two major distribution patterns, represented by the ones based on the R1 and M1 biclusters (Figure 8C and D). All other clusters generated a distribution similar to M1 (M2–M4; R2–R4, data not shown). Whilst the distribution of samples with different mutations in the M1 bicluster clearly separated VHL mutants from both a group of sporadic and RET mutant tumors based on the average expression of clustered genes, SDHx mutants were rather separated by their low correlation. Thus, the R1 cluster distribution, where VHL and SDHx mutant tumors were clearly separated fork patterns, was representing better the biclustered gene group of which the expression determines the differences between the VHL and SDH deficiency driven phenotype. GO analysis of the gene group most highly correlated with the pattern revealed several enriched GO terms, indicating large scale changes in cellular phenotype. In order to determine whether mitochondrial metabolic pathways are implicated in the adaptation, we have selected for further analysis a custom gene group of a particular pathway, the TCA cycle. Cardaci *et al*. (57) demonstrated that metabolic adaptation to SDH deficiency relies on pyruvate carboxylase generated aspartate through elevated flux through glutamic-oxaloacetic transaminase (GOT1, cytosolic, GOT2 mitochondrial isoforms), associated with malate production through malate dehydrogenase (MDH1 cytosolic, MDH2 mitochondrial isoforms). Analysis of the correlation strength of TCA enzymes is shown in Figure 8E. Importantly, while most TCA cycle enzymes in the VHL mutants samples appear to be suppressed as compared to sporadic and RET mutant samples (M1 bicluster, lower fork), probably reflecting the activation of the HIF1α pathway, differential expression of specific sets of TCA enzymes between SDHx and VHL samples is revealed by the R1 bicluster. The expression of the enzymes PC, GOT and MDH is highly correlated with the SDHx fork (R1 bicluster, upper fork), indicating their central role in gene expression mediated adaptation to SDH deficiency. These results wholly predict the metabolic phenotype characterized by Cardaci *et al*. (57), as depicted in the scheme in Figure 8E. Crucially, comparison of average TCA cycle enzyme gene expressions between the SDHx and VHL mutant samples by standard Limma differential gene expression analysis could not faithfully reveal the pattern (Supplementary Figure S7), likely due to the noise caused by the inclusion of a small number of outlier samples which do not show high correlation with the bicluster pattern. This analysis demonstrated the advantage of using correlation based sample ranking by MCbiclust in identifying the key elements contributing to the specific gene expression pattern.

## DISCUSSION

To tackle problems of biclustering, more recent biclustering methods have attempted to introduce some kind of bias to their algorithm to make the searching for relevant biclusters in the NP-hard problem more efficient. BicNet (59) uses a pattern based algorithm method to find biclusters based on interactions in sparse data networks such as those representing biological networks. The work by Nepomuceno *et al*. (60) meanwhile inputs biological annotation data into the fitness function to identify biclusters. Most biclustering methods would use this annotation data to validate found biclusters (37), Nepomuceno *et al*. (60) however argues that their approach is less likely to identify biclusters composed of co-expressed genes that are the result of independent activation.

It is important to note that some biclustering methods (61) seek biclusters where not just the genes but also the samples are highly correlated. A bicluster with highly correlating samples however need not have highly correlating genes, for instance a group of replicate samples will be highly correlated with each other yet their genes would be expected to randomly vary around a mean value leading to close to zero correlation between the genes. Thus seeking biclusters with highly correlated samples would bias towards finding samples that are very similar but with no significant change in gene expression between them. Additionally biclusters composed of large number of genes with significant alterations are of interest since they represent large patterned changes in transcriptional programs within the cell. Such changes are well known to occur, an example would be metabolic adaptations such as up-regulation of mitochondrial biogenesis in response to cold (62).

In addition to this theoretical considerations, the direct comparison of MCbiclust with other common classification and data reduction methods revealed important differences. On the one hand, MCbiclust bears some similarity to Weighted Gene Co-expression Network Analysis (WGCNA) (63); in WGCNA there is a concept of modules, that are clusters of highly interconnected genes with high absolute correlations, the difference is that in WGCNA these correlations are across all the samples while in MCbiclust they are found only in a subset. In addition to this other workings of WGCNA also have some similarity to MCbiclust. The correlation vector in MCbiclust can be compared to WGCNA’s intramodular connectivity measure; the module eigengene in WGCNA is defined as the first principal component and is considered a representative of the gene expression profile in a module and is very similar to what is done in MCbiclust. However, despite these similarities WGCNA and MCbiclust are fundamentally different in that WGCNA studies global co-expression across all samples while MCbiclust aims to find co-expression across subsets of samples. This feature makes MCbiclust more proficient in classifying samples according to gene expression patterns, a much sought after characteristics of algorithms aimed to stratify large amount of samples. In the same way when compared to dimensional reduction techniques such as PCA, ICA (64) or t-SNE (65) the aims of these techniques are fundamentally different to that of MCbiclust. The biclusters found in MCbiclust not being universal across all samples are not expected to be much use in dimensional reduction, while dimensional reduction methods seeking universal patterns would not be expected to identify individual biclusters.

MCbiclust outperforms other biclustering methods in terms of identifying large biclusters. The approach presented in this paper offers a new paradigm in the analysis of gene expression levels. This approach is pattern-centric, with large numbers of significantly co-regulated genes being sought unsupervised in a minority of the samples, once found both genes and samples can be ranked by how strongly an individual gene is being co-regulated in the pattern or how strong is this co-regulation in the sample. It has been demonstrated that the patterns it finds are biologically relevant and meaningful and it has great potential use in the analysis of transcriptomic datasets and classifying samples in a novel, biologically relevant way, according to their large-scale gene transcription pattern.

A simple example for improving transcriptome analysis stems from the finding of a DNA replication effect hidden in the gene expression data within the M^3D^*E. coli* data set (Figure 5D and E). By revealing this effect, MCbiclust now makes it possible to normalise for it, e.g. in order to remove bias, allowing analysis of other gene sets with low signal strength.

Similar improvement in analysis can result from the finding in the third E3 bicluster. It is unusual in that a single sample with extreme global differences in gene expression has driven the formation of this bicluster. This sample was from an original study involving 16 Affymetrix arrays with two replicates over eight conditions (66). Examining the images of the raw Affymetrix CEL files reveals that this sample (MGD1_t0_A.CEL) has very weak intensities over most of the chip compared to its replicate (MGD1_t0_B.CEL) and other samples within this study. This has probably arisen due to some problem with sample preparation since other aspects of the chip (such as spike-in concentration gradients) are normal. RMA normalization of this chip has brought these low gene expression values in line with other chips, but the normalization in turn causes a number of genes (mostly intragenic) to have abnormally high values. The resulting large-scale transcriptional pattern is what MCbiclust has detected within E3, and although not biological in nature, it does show the methods impressive power to find a single chip that has either sample or normalization issues within a very large data collection; thus potentially of use for data cleansing large –omics data collections. Interestingly Figure 5C shows a few other samples within this data collection that potentially have similar sample preparation issues but not as extreme as this sample.

An intriguing feature of MCbiclust is that by creating *PC1 versus ranking* plots, the distribution and classification of samples can be better understood. Thus MCbiclust first discriminates samples according to the strength of correlation of a specific gene set, thus recognizes classes of samples with high and low correlation, indicating that a specific gene expression pattern is being regulated or not in a specific class. However, since this regulation can be either positive or negative (creating anti-correlation patterns, see Figure 4), samples with higher expression of a subset of genes from the bicluster are clearly separated from samples having the gene set suppressed. This next level of classification, e.g. in the Mito bicluster, most probably reflects mitochondrial biogenesis (high in the upper fork samples), which is either activated or suppressed according to the metabolic needs of tumors (67). Such classifications have high chance of applicability both in discovery or clinical science based on gene expression data. For instance, since the correlation vector of the bicluster is known, expression of each gene of the genome, even outside the bicluster, can be correlated with it. Thus, a correlation value can be associated with any gene, allowing the analysis of other cellular processes either acting upstream (e.g. master gene regulators of large gene sets or genetic changes), or downstream of the action of the bicluster. Of clinical relevance, correlation with clinical pathological phenotypes, differences in pharmacological sensitivity as shown in Figure 7C and D, or differences in metabolic phenotypes (Figure 8C and D) can be revealed by MCbiclust, suggesting that it may be possible to use these biclusters in future for prediction of the phenotype of tumors, potentially informing on drug sensitivity or serve as base to find new pharmacological targets. Interestingly, similar biclusters such as Mito and R1 in the CCLE dataset, or the series of mitochondrial and random biclusters in the pheochromocytoma/paraganglioma dataset, predict slightly different tissue distributions (compare Figure 6C and D, not shown for the latter example), indicating that the cellular phenotype is somewhat sensitive to small changes in the correlation vectors and the genes involved. In addition, the two CCLE biclusters predicted differential sensitivity between upper and lower fork samples to a common set of drugs (Figure 7D), but bicluster-specific drugs have also been found.

Another feature, and a current potential weakness of the current method is that a few biclusters with strong correlation signals will dominate the results. This feature on the one hand ensures the discrimination of robust biclusters from noise, but at the same time might exclude some further biclusters to be found. Probably this characteristics is responsible for MCbiclust missing two synthetic biclusters (Figure 2). By enriching the algorithm, we need to build an adapted version of MCbiclust that can be enabled to also identify weak signaled biclusters. In addition, apart from further developing the mathematical system, it will be of value to seek applications across all areas of gene expression research, from gene network regulation to biomarker discovery.

## Supplementary Material

Supplementary Data
