# Supplementary material for: MCbiclust: a novel algorithm to discover large-scale functionally related gene sets from massive transcriptomics data collections
Source: Nucleic Acids Res. 2017 Jul 14;45(15):8712–30. doi: 10.1093/nar/gkx590 (PMC5587796; doi:10.1093/nar/gkx590)
Supplement: Supplementary Data [file gkx590_supp.zip › nar-01701-z-2017-File011.pdf]

Supplementary Methods

Bicluster Quality Metric: Let I be the subset of genes and J be the subset of samples within a bicluster. MCbiclust judges a bicluster's quality using the mean absolute average value of the gene-gene Pearson's correlation coefficient matrix of the subset of probes calculated from the subset of samples:

$$\alpha = \frac{1}{|I|^2} \sum_{i \in I} \sum_{k \in I} abs(C_{i,k}^{J})$$

A high value for α indicates that the probes in the gene set I are strongly co-regulated across the samples J. Note that as α is calculated using the absolute values of the correlation matrix, these genes may be either in correlation or anti-correlation with each other.

Initial selection of seed genes: The algorithm is initially 'seeded' with around 1000 genes related to the biological process that the user is interested in (for instance MitoCarta genes if the user is interested in mitochondrial processes) or, alternatively, 1000 random genes if the user wishes to detect large correlated genes sets within subsets of the samples.

Heuristic to find biclusters: A stochastic greedy search is used to find a small number of samples, n, which have a high value of α. In all runs of the algorithm n = 10 and the stochastic greedy search was run for 1000 iterations of replacing a single one of the 10 samples and keeping the new sample set if the resulting value of α was higher. In this way MCbiclust finds a small 'seed' bicluster containing a relatively large number of genes (~1000) but small number of samples n. Once found this bicluster 'seed' can be extended. First however α is further maximised by selecting only a subset of genes in the bicluster.

Pruning the bicluster genes: After finding 10 samples α is further maximised by selecting a subset of the genes. Hierarchical clustering is used to divide the genes into m groups, with m = 8 for all runs of MCbiclust. The α values for these 8 groups of genes against the 10 samples are then calculated. The genes in the groups that have a higher α value than the original are retained while those with a lower value are removed. The number of groups m should not be too large or too small a value, if it is too small then too many genes may be removed, and if it is too large then the correlations between many genes will not be included in the calculation for α, a typical value of m is 8. At this point the full 'bicluster seed' has been found, and what is left to do is to extend it to find addition samples and genes that belong to the bicluster.

Extending the bicluster - samples: The remaining samples can be ranked by how well they preserve the correlation. That is the (n+1)th sample is the sample added to the n previous samples that results in the highest value of α, this can be continued until all the samples in the data set are ranked.

Extending the bicluster - genes: There may be many genes not in the original gene list chosen that could be part of the bicluster. Due to the large number of genes in a gene expression matrix it is not practical to rank them, as was done for the samples. Hierarchical clustering is used to divide the genes within the original gene list into 8 groups, and the group with the highest α value against the top 10 samples is chosen as being 'strongly representative of the bicluster'. The average expression of these genes across the 10 samples is calculated, with this a vector of every gene's correlation to this average expression, describing its relation to the bicluster, can be calculated. This vector is known as the correlation vector (see Supplementary Fig. S1). This correlation vector describes the correlation of every gene measured to the bicluster. Genes with high absolute values within the correlation vector can thus be considered to be co-regulated with the genes in the bicluster and hence belong to the extended bicluster.
After a bicluster has been found with samples ranked and the correlation vector calculated, the bicluster needs to be interpreted. This can be done by studying the samples or genes.

Analysing the bicluster - samples: Using the genes found to be highly correlated, principal component analysis (PCA) is run on the top 10 samples. The calculated eigenvectors from the principal component analysis of the top 10 samples are then used to fit the first principal component (PC1) value for every sample using least-squares. In this way the correlation of the highly correlating genes is summarised by the values of PC1. When plotting PC1 against the sample ranking, it is typical to see a fork like pattern, with samples at the beginning of the ranking separated into an upper and lower fork (see Fig. 3 and Supplementary Fig. S5). In a post-processing step the upper and lower fork samples can then be compared to additional information in the data set to see if they divide it in biologically relevant ways (see Figs. 4-6).

Analysing the bicluster - genes: For post-processing gene set enrichment analysis can be run on the correlation vector, for full description of this see the later section *Gene set enrichment analysis.*
The output of MCbiclust so far give a ranked list of samples with associated PC1 values along with all genes having a correlation vector value, post processing of this output can associate PC1 values and sample rankings with biological phenotypes and study gene set enrichment within the correlation vector. There is however one more aspect to the output from MCbiclust, instead of a ranked list of samples and genes it may be required to have a threshold to decide on which genes and samples belong to the bicluster and which do not.

Thresholding the bicluster: To determine which genes and samples are in the bicluster and which are not, k-means clustering is used to divide the genes into 2 groups using their absolute correlation vector values, one group regulated by the bicluster and one not. For samples the last 10% of ranked samples is assumed to not belong to the bicluster and the

range of PC1 scores for these samples is used to judge whether a sample is or is not part of the bicluster.

As the initial heuristic search is stochastic, different runs produce different results. To find all the biclusters in a data set MCbiclust must therefore be run multiple times. Instead of repeating all steps of the analysis for all runs, once the correlation vectors have been calculated they can be compared to save computation time, with only the 'distinct biclusters' found being further analysed.

Multiple runs: When attempting to find multiple biclusters the procedure for calculating the output of MCbiclust is slightly different. For each run started with a different random seed only the correlation vector is calculated. Often these correlation vectors are highly similar and perhaps even identical, instead of the full MCbiclust output for all these runs, the number of distinct correlation vectors is calculated and only these have the full MCbiclust output calculated. The number of distinct biclusters is found by silhouette width analysis where 1 minus the absolute correlation between two correlation vectors is used as the dissimilarity score. This method however will always give 2 or more optimum clusters, a synthetic correlation vector containing random noise can be added to the data to check if the real data are better as one single cluster. The average correlation vector of each distinct bicluster is calculated, samples are ranked by taking the top 1000 genes in this average correlation vector and selecting the distinct seed as the set of 10 samples out of all identified that has the maximum α for these top genes. Using the average correlation vector, and the 10 samples in the chosen seed for each distinct bicluster the rest of MCbiclust output can be calculated by the previously described procedure.

Gene set enrichment analysis: The Mann-Whitney or the Wilcox Rank Sum test is used to test for significance between (i) genes in a gene set specific to a pathway, GO category or other custom feature (e.g. intergenic location) and (ii) genes that are not in this set, by examining the values of the correlation vector. The Mann-Whitney test is non-parametric which is used since the distribution of correlation vector values are bounded between -1 and 1 and are very often bimodal, and therefore parametric tests such as the t-test are unsuitable. For the genes in a particular pathway, the values of the correlation vector for those genes are compared against the values of the entire correlation vector. The Mann-Whitney test works by assigning a numeric rank to all the genes in the correlation vector, the sum of the ranks of the genes in the pathway of interest is then calculated and from this a statistic U can be calculated that is used to find p-values.

Optimising bicluster parameters: For optimisation of the parameters, 10 synthetic data sets were randomly generated using FABIA as before, with each data set containing 8 non-overlapping biclusters.

For MCbiclust the following parameters were varied: 1) seed.size = the seed size (5, 10, 20,

50); 2) iters = the number of iterations (1000, 2000, 5000, 10000); 3) gsplits = the number of groups to divide the genes into when calculating the correlation vector (4, 8,16) ; 4) alpha = a parameter between 0 and 1 (0, 0.2, 0.4, 0.6, 0.8) used in selecting the number of samples in the bicluster, with values close to 1 being less strict in accepting samples as members of the bicluster. The results of this can be seen in Figure S3.

Each combination of these parameters was tested using grid search and the optimum combination found to be seed.size = 10, iters = 2000 and gsplits = 4, alpha=0.8. However the main variation comes from the seed size, a low seed size of 5 results in a large number of biclusters being identified, this is likely due to the increased noise in the correlation matrix of only examining 5 samples while searching for an initial seed. A high seed size of 50 however leads to too few biclusters being identified, implying that biclusters with weaker signals are missed. Both the seed size of 10 and 20 found similar numbers of biclusters however the biclusters found using a seed of size 10 had a much higher consensus score to the actual biclusters than those found with a seed size of 20. This indicates that if the seed size is too large the biclusters are more likely to represent optimised local maxima rather than global maxima.

There is little overall effect from the number of iterations except suggesting that 1000 iterations is enough to find the local maxima under nearly all parameters. The only exception being when the seed size is 5, in which increasing the number of iterations leads to the quality of the biclusters found decreasing. There is also little effect from altering parameter c, the number of groups to divide the genes into. Finally increasing the sample thresholding parameter alpha increases performance.

For ISA the parameters thr.row and col.row were optimised by grid search with both being selected a value between 0 and 4 (0, 0.5, 1, 1.5, 2, 2.5, 3, 3.5, 4). The optimum parameters were chosen as thr.row = 0.5 and thr.col = 1.5.

For FABIA the parameters alpha, spl, spz and random were optimised by random search. Random search was used due to the large search space, parameters were randomly generated 100 times and applied to each of the 10 different synthetic data sets. The optimum parameters were alpha = 0.2134905, spl = 1.942457, spz = 1.367281 and random = -0.71584. None of the parameter values resulted in a consensus score higher than 0.06

Designing additional synthetic data sets: Synthetic data was created to investigate the effect of noise, bicluster size, number of biclusters in the data set and whether different biclusters had overlapping samples. In order to do this three different sets of data were created to investigate different effects. These were as follows:

1. Data sets investigating the effect of noise and size, each containg a single bicluster of various sizes and levels of noise.
2. Data sets investigating the effect of different number of biclusters with overlapping samples.
3. Data sets investigating the effect of different number of biclusters with non-overlapping

samples.

For the noise and size datasets the synthetic data was generated as previously with an adapted version of the method described in FABIA, this version was the same as used in the preliminary synthetic data generation but with an additional step where all but one of the biclusters and their genes were randomly resampled for each sample in the bicluster thus destroying the structure of the bicluster and creating a relevant bicluster. The remaining single bicluster varied in size (25 samples x 150 genes, 50 samples x 300 genes, 100 samples x 600 genes) and had 4 different levels of noise (Gaussian zero mean noise with standard deviation on data matrix being either 0.01, 0.2, 0.4 or 0.8).

For the overlapping samples data sets the synthetic data was generated with the default method described in FABIA, slightly adapted so that each bicluster was normalised to its row (sample) mean, with each bicluster containing on average 95 samples and 451 genes with differing numbers of biclusters (2,4,8,16).

For the non-overlapping data sets this was done as previously described for the preliminary synthetic data using an adapted version of the method described in FABIA, with biclusters containing on average 95 samples and 451 genes though with differing numbers of biclusters (2,4,8 and 16).

For every condition 10 datasets were generated with different random seeds.

FABIA and ISA applied to the E. coli dataset: FABIA and ISA were both applied with the parameters previously identified as optimum with FABIA being run to identify 8 bicluster. ISA using optimum parameters identified 10 biclusters. The Munkres algorithm was used to identify which of the biclusters found using FABIA and ISA were most similar to E1, E2, E3 (see main text) identified by MCbiclust. The comparison of these matched biclusters is given in Table S2 showing that FABIA and ISA, while identifying some biclusters with many shared genes/samples with those in MCbiclust, are finding much smaller biclusters. These biclusters being smaller than those found with MCbiclust and if containing many highly correlated genes would be expected to have a higher correlation score, however this is not the case. With ISA the gene score and sample scores associated with each bicluster can be examined and it can be seen how these relate to the correlation vector and PC1 respectively. For the matched E1 bicluster the samples exclusively have high PC1 values indicating membership of the upper fork, while the genes mostly have high positive correlation vector values, this can be seen in Figure S8A. An even stronger effect can be seen in the E2 match bicluster and can be seen in Figure S8B. This indicates that in these cases ISA only succeeds in finding a subset of the MCbiclust biclusters.

Guide to setting parameters for MCbiclust: The key parameters to set in MCbiclust are the sample seed size, number of iterations and number of initial genes to run MCbiclust on.

Of these the sample seed size is perhaps the most critical, if it is too large the possible search space will be too big but if it is too small the found biclusters will not be robust to noise and

random variations in one or two samples. When optimising MCbiclust (see previous section) and in practice we have found the ideal size of a sample seed to be 10, with this biclusters can be found efficiently and be robust to noise.

Ideally the higher the number of iterations to find the initial sample seed the better, however to save computational time it is useful to find a good minimum number that still finds quality biclusters. In practice, we find that 1000 iterations is often enough to identify quality biclusters, in the MCbiclust the optimum number of iterations tested was 2000 but the difference between all was minimal.

The number of initial genes to run MCbiclust on is limited by computational efficiency. MCbiclust computes many correlation matrices, in practice we find that an initial gene set of sizes around 1000 is sufficient to find large biclusters and can be run on MCbiclust in a manageable time.

Other parameters have less of an effect in the output of MCbiclust, before calculating the correlation vector, genes not well correlated to the bicluster are removed by using hierarchical clustering to divide the genes into *m* groups. Later in the MCbiclust pipeline the correlation vector is calculated by again dividing the genes into *m* groups and choosing the group that most strongly represents the bicluster. Optimising of MCbiclust showed that changing these parameters have little effect over the outcome, unless the sample seed size was small. The default value is 8 and this has been shown to work well in practice.

The final parameter of importance is the one used to threshold the bicluster samples, alpha, this must be a value between 0 and 1 and represents how strictly a sample is accepted into the bicluster. When alpha = 0, the first sample to fall in the range of the last 10% ranked samples and all samples after that are excluded. When set to alpha = 0.5 the first sample to fall within the inter-quartile range of the last 10% ranked samples and all samples after that are excluded. Optimising MCbiclust shows that often a less strict approach is better with higher consensus scores being achieved with a value of alpha = 0.8.

Supplementary Table and Figure Legends

Supplementary Table 1. Previously established methods used in benchmarking.

Supplementary Table 2. Table comparing biclusters found from FABIA and ISA with the three biclusters (E1, E2 and E3) found with MCbiclust discussed in the main text. The number of significant GO terms was calculated using the Mann-Whitney test applied to the correlation vector in the case of MCbiclust and the ISA gene scores in the case of ISA. In the case of FABIA the Mann-Whitney test was applied on a vector 1's and 0's with 1's representing membership of the FABIA bicluster.

Supplementary Table 3. Size of the synthetic biclusters used in the preliminary analysis.

Supplementary Figure S1. Schematic showing the process of calculating the correlation vector in MCbiclust. MCbiclust is used to find the core bicluster of the top 10 samples and the selected gene set. Hierarchical clustering is used to divide the genes into 8 groups, and the group with the highest α value against the top 10 samples is chosen as the gene set determining the bicluster. The average expression of these genes across the 10 samples is calculated. Finally, the correlation vector is calculated, representing the correlation of the expression of each gene across the investigated samples to this average expression and hence to the bicluster. Since the correlation vector describes the correlation of every gene measured to the bicluster, it can be used as an indicator to find co-regulated gene sets.

Supplementary Figure S2. Relative operator curve (ROC) plots of the outputs of the different biclustering algorithms compared to the known synthetic biclusters (S1-8). Since the output of MCbiclust is a ranking for genes and samples, a complete curve can be produced, while other biclustering methods return a definite bicluster thus these are represented as points. Points are also used to mark the output of the MCbiclust threshold method for defining a bicluster (MCb_thr) and the optimum bicluster (MCB_opt) according to the Jaccard index that can be produced out of the MCbiclust rankings.

Supplementary Figure S3. Comparison of different parameter settings on the consensus score for runs of MCbiclust on synthetic data. Number of iterations (A), gene groups splits (B) and threshold paramteres (C) plotted against consensus scores using different seed sizes (5-50 samples, color scaled). The most crucial parameter is the seed size which is optimum with a value close to 10.

Supplementary Figure S4. Parameter optimization for ISA (**A**) in two and FABIA (**B**) in five dimension plots to obtain the highest consensus score.

Supplementary Figure S5. The relationship between PC1 and average gene expression of gene groups in the samples determining a bicluster. As an example, ranked sample plots of

the M1 bicluster found with MCbiclust in the synthetic data, representing the S5 synthetic bicluster. **A.** PC1 versus sample ranking plot, showing the typical distribution of the bicluster into upper and lower forks (indicated with arrows). Samples belonging to the upper and lower forks are represented by red and blue circles, respectively. **B.** Average gene expression of gene group 1 versus sample ranking plot. **C.** Average gene expression of gene group 2 versus sample ranking plot. The red and blue annotation of upper and lower fork refers to as it is described in **A**. Gene groups 1 and 2 were determined from hierarchical clustering the correlation of all genes in the bicluster. Samples of the bicluster belonging to the upper and lower fork are determined after settling the ranking threshold (shown in **A**), as described in the Supplementary Methods.

Supplementary Figure S6. All unique biclusters found by MCbiclust in the CCLE dataset using Mitocarta (Mito) and random (R1, R2) initial gene sets. **A.** Heatmap of correlation matrix of correlation vectors for MCbiclust runs on the CCLE data with Mitocarta initial gene set and silhouette analysis plot grouping correlation vectors into one main cluster (Mito). **B.** Heatmap of correlation matrix of correlation vectors for MCbiclust runs on the CCLE data with 1000 random initial gene sets and silhouette analysis plot grouping correlation vectors into two clusters (R1 and R2). **C.** Gene-gene correlation matrices R1 and R2 produced from MCbiclust following identification of a subset of samples using random gene sets. Correlation matrices before gene set pruning with Hicor are shown.

Supplementary Figure S7. Additional analysis of gene expression patterns in pheochromocytoma and paraganglioma tumour samples with germline mutations. A, B. Correlation vectors of the biclusters found by MCbiclust in the set of 239 pheochromocytoma and paraganglioma tumour samples. Correlation vectors are divided into unique bicluster groups M1-4 (**A**) and R1-R4 (**B**) from the output of the silhouette analysis. The silhouette plot of the optimum number of clusters is shown as chosen by maximizing the average silhouette width of all the correlation vectors. For correlation heatmaps of the biclusters see Fig. 8. **C**. Venn diagram of metabolism related GO-terms discovered by comparing differentially expressed genes between the R1 bicluster upper and lower forks (violet), all SDHx and VHL mutated samples (green) and SDHx and VHL samples not present in the R1 bicluster forks (pink). Intersection of all sets uniquely identify ‘Alanine, asparate and glutamate metabolism’, reflecting experimental findings (see Fig. 8). **D**. Comparison of TCA cycle enzyme gene expressions (named in left column) by MCbiclust correlation vectors in the R1 bicluster (as shown in Fig. 8) and standard Limma analysis of log fold changes between all SDHx and VHL samples. While MCbiclust identifies a set of enzymes in a set of samples with high correlations (group of 8 enzymes > 0.8), while Limma highlghts one candidate enzyme.

Supplementary Figure S8. A comparison of the ISA gene scores with the correlation vector as well as the ISA sample scores with the PC1 values for the MCbiclust biclusters and their matched ISA biclusters. Samples were scored by ISA, and the parameters were plotted against MCbiclust correlation vector and PC1 in three real data (E. coli) biclusters (E1-3).

Method	Description	Software
MCbiclust	The method developed in this paper, outputting a ranked list of the genes/probes and samples.	Run with R package 'MCbiclust'
FABIA (41)	Factor analysis for bicluster acquisition.	Run with R package 'fabia'
FABIAS (41)	A variation of the FABIA method using a different prior distribution in the model.	Run with R package 'fabia'
biMax (38)	Assuming a binary data model, uses a fast divide and conquer strategy to find biclusters, originally designed as a reference method to compare different biclustering techniques.	Run with R package 'biclust'
CC (28)	Landmark method that originally applied biclustering methods to gene expression data, strategy is to find biclusters which minimise the mean square residue.	Run with R package 'biclust'
Plaid (42, 68)	Biclusters form layers that are superposed to form the data matrix, the algorithm aims to minimise the sum of square errors matching the model to the data.	Run with R package 'biclust'
ISA (43)	Iterated Signature Algorithm, designed to work on very large datasets, and decomposes them into modules.	Run with R package 'isa2'
FLOC (41)	Flexible Overlapped biClustering, uses a stochastic iterative greedy search, to find possible overlapping biclusters.	Run with R package 'biCARE'
QUBIC (45)	Qualitative biclustering algorithm is a non-metric method that uses ideas from graph theory to find biclusters.	Run with R package 'rqubic'
CPB (46)	Correlated Patterns Biclustering, a method utilising Pearson's correlation as its quality measurement score.	Run with python script
CTWC (47)	Coupled Two-Way Clustering used in conjunction with superparamagnetic clustering algorithm (SPC).	Run with windows software

Supplementary Table 1

MCB	Jaccard Index	Probe set size	Probe Intersection	Sample set size	Sample Intersection	Correlation Score	GO terms sig
E1	-	4823	-	375	-	0.4461589	175
E2	-	4700	-	211	-	0.5453846	25
E3	-	6106	-	193	-	0.2502874	196
**FABIA**	**Jaccard Index**	**Probe set size**	**Probe Intersection**	**Sample set size**	**Sample Intersection**	**Correlation Score**	**GO terms sig**
E1	0.06882154	1968	1918	223	89	0.3043734	36
E2	0.04387621	535	535	170	106	0.4161746	4
E3	0.002523775	256	199	276	34	0.3543747	47
**ISA**	**Jaccard Index**	**Probe set size**	**Probe Intersection**	**Sample set size**	**Sample Intersection**	**Correlation Score**	**GO terms sig**
E1	0.02976302	2131	1595	43	38	0.2803084	112
E2	0.17225216	2604	2019	114	101	0.3268816	26
E3	0.05361609	2290	1860	53	39	0.246038	133

Supplementary Table 2

	Gene set size	*Sample set size*
S1	472	116
S2	467	152
S3	476	107
S4	492	165
S5	493	136
S6	515	125
S7	501	104
S8	454	154

Supplementary Table 3

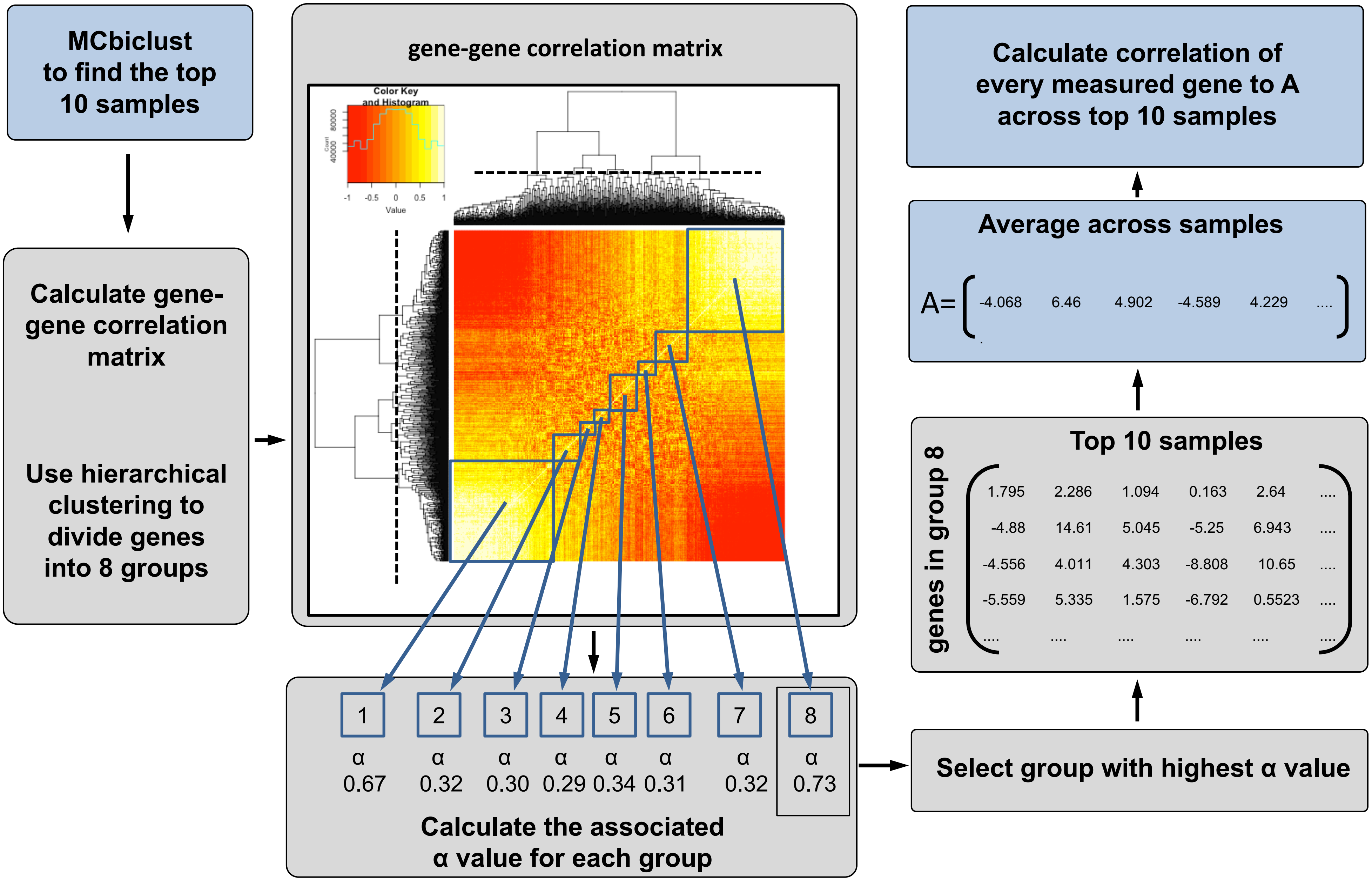

Supplementary Figure S1

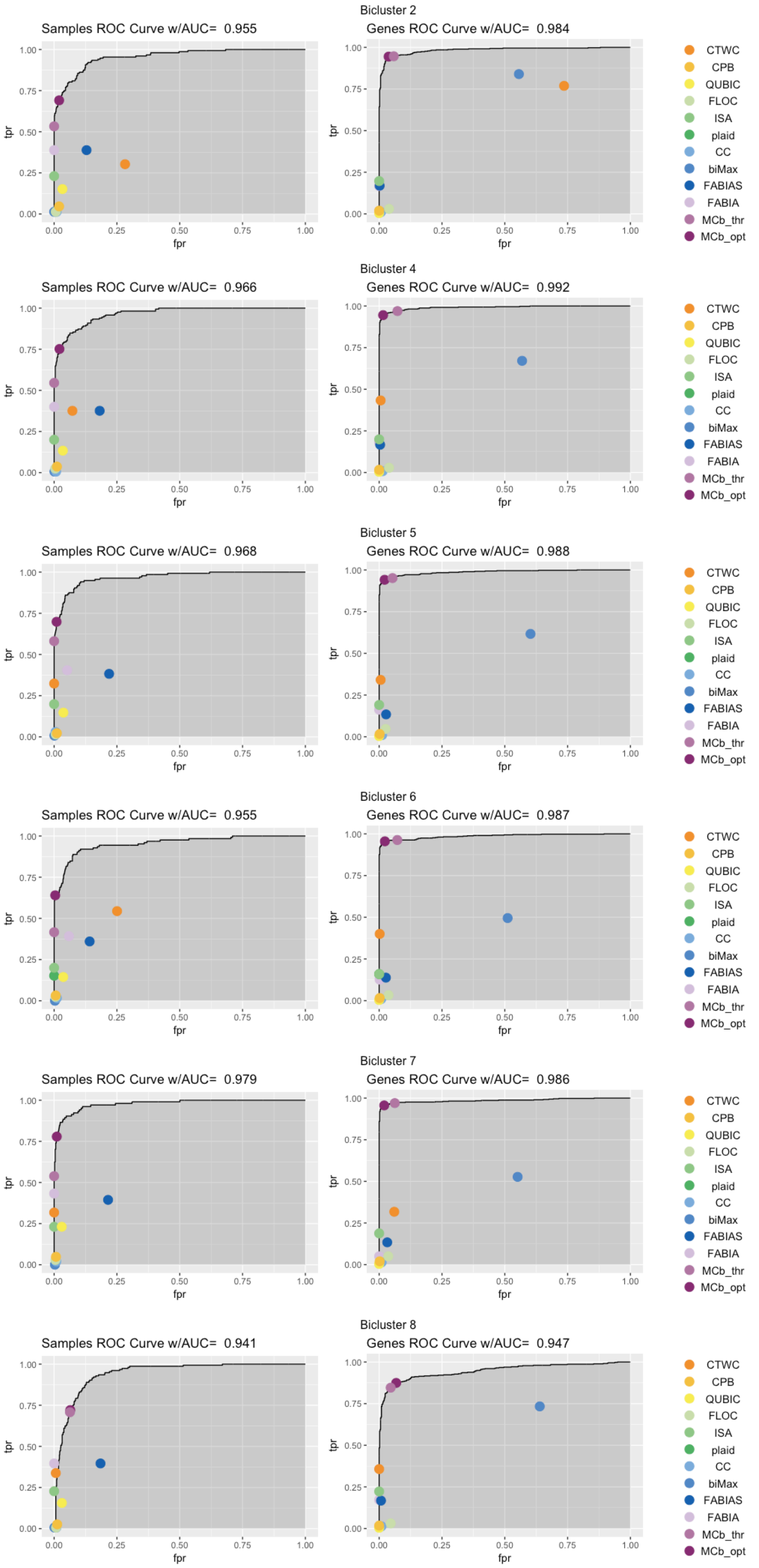

Supplementary Figure S2

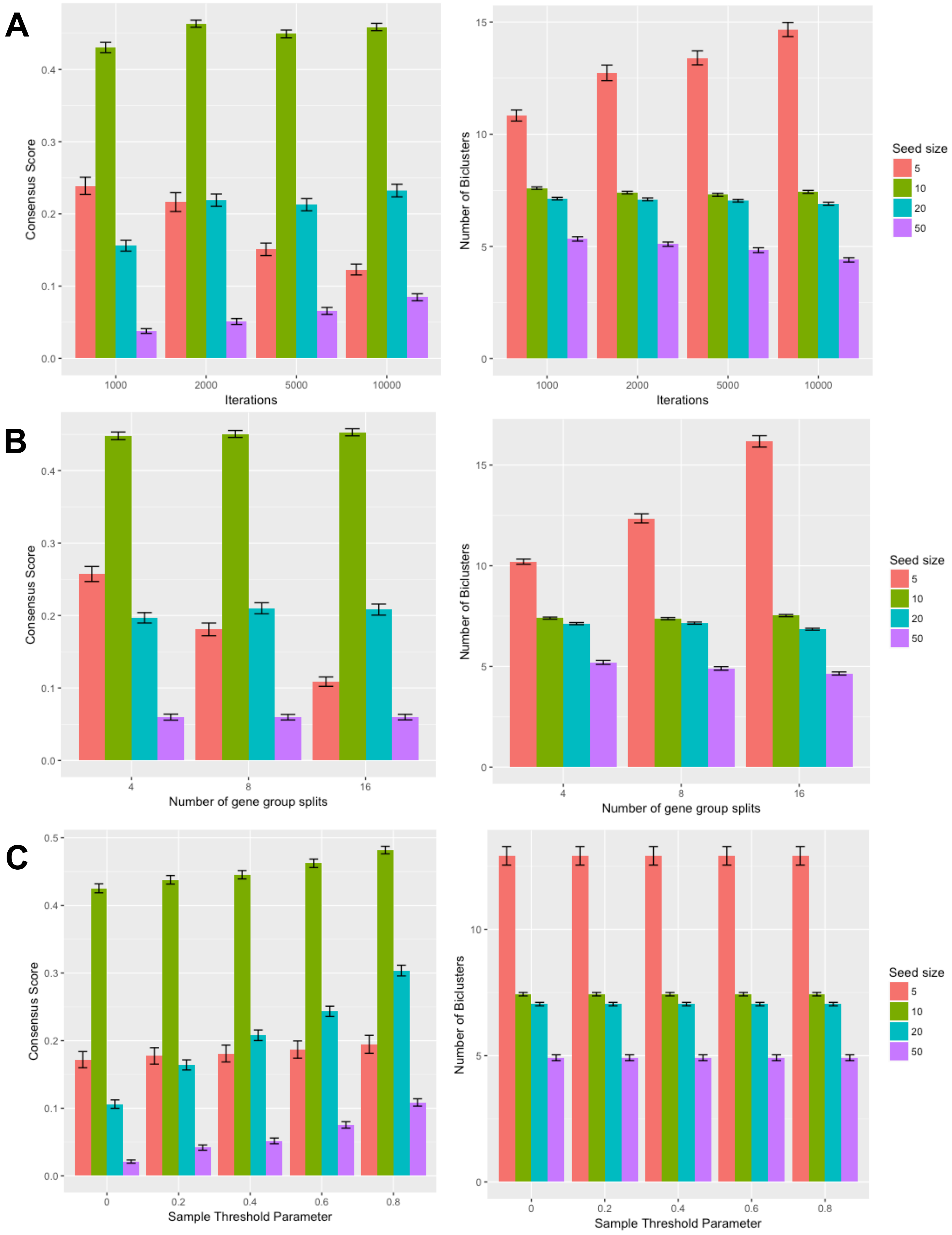

Supplementary Figure S3

A

B

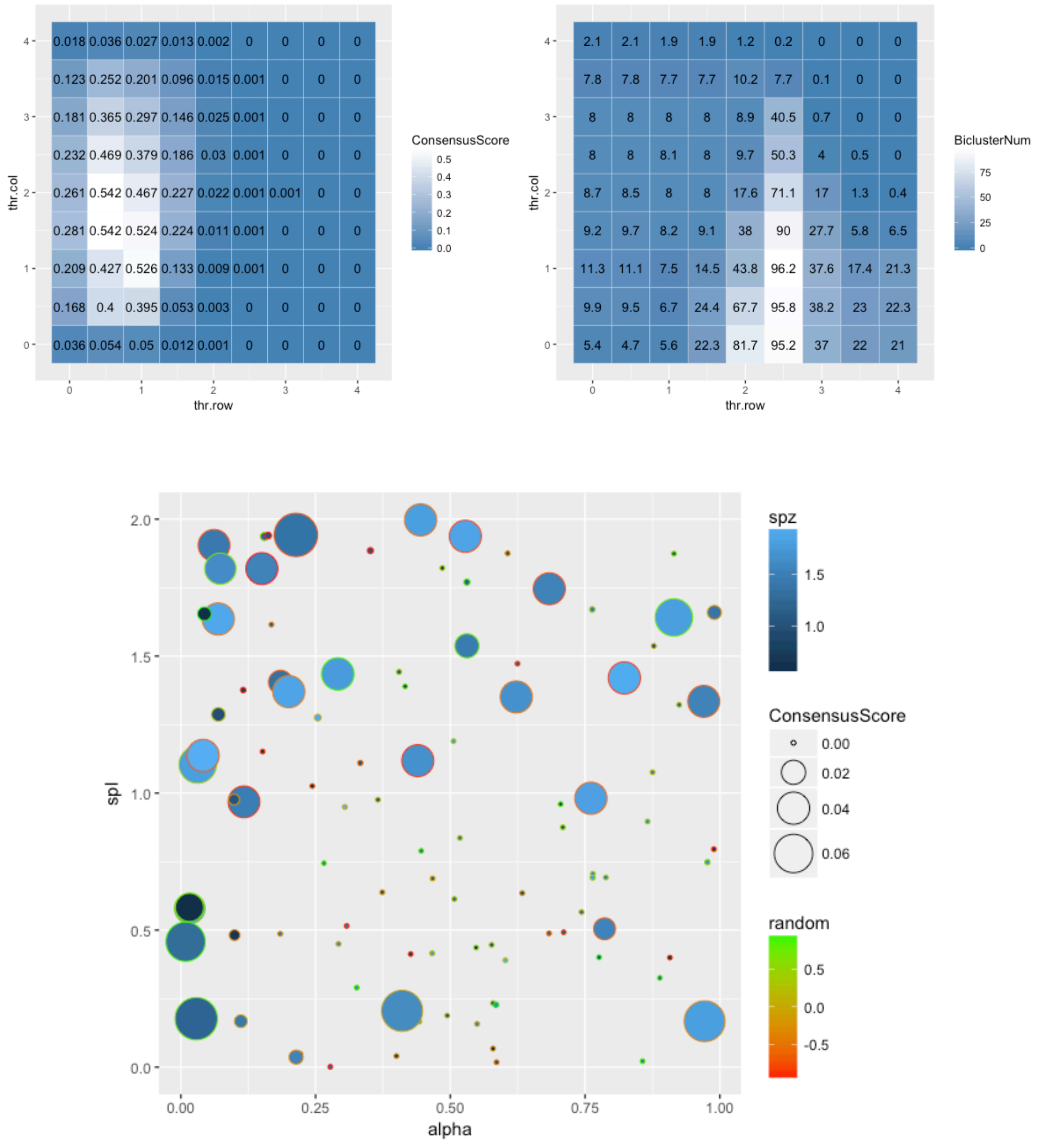

Supplementary Figure S4

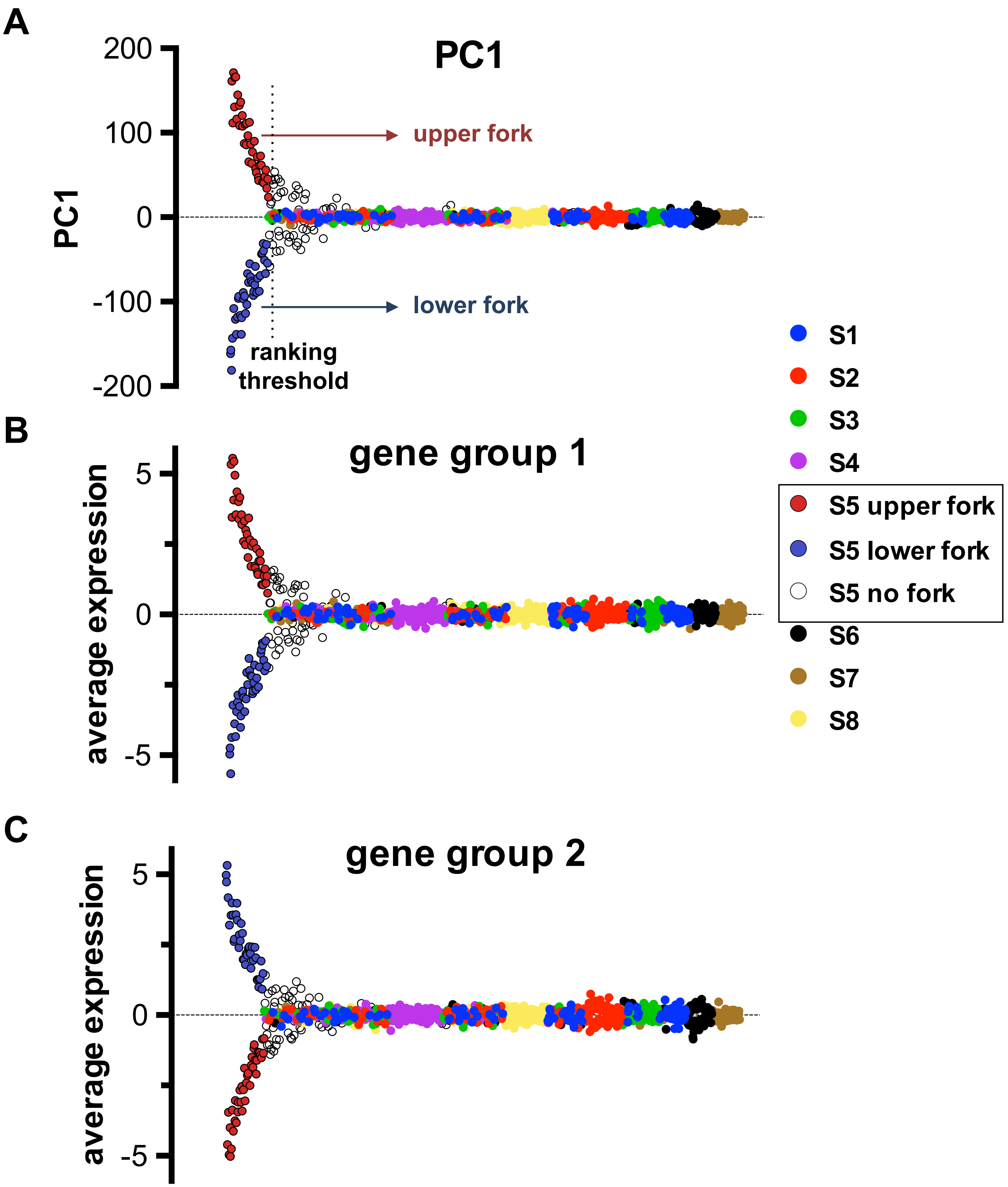

Supplementary Figure S5

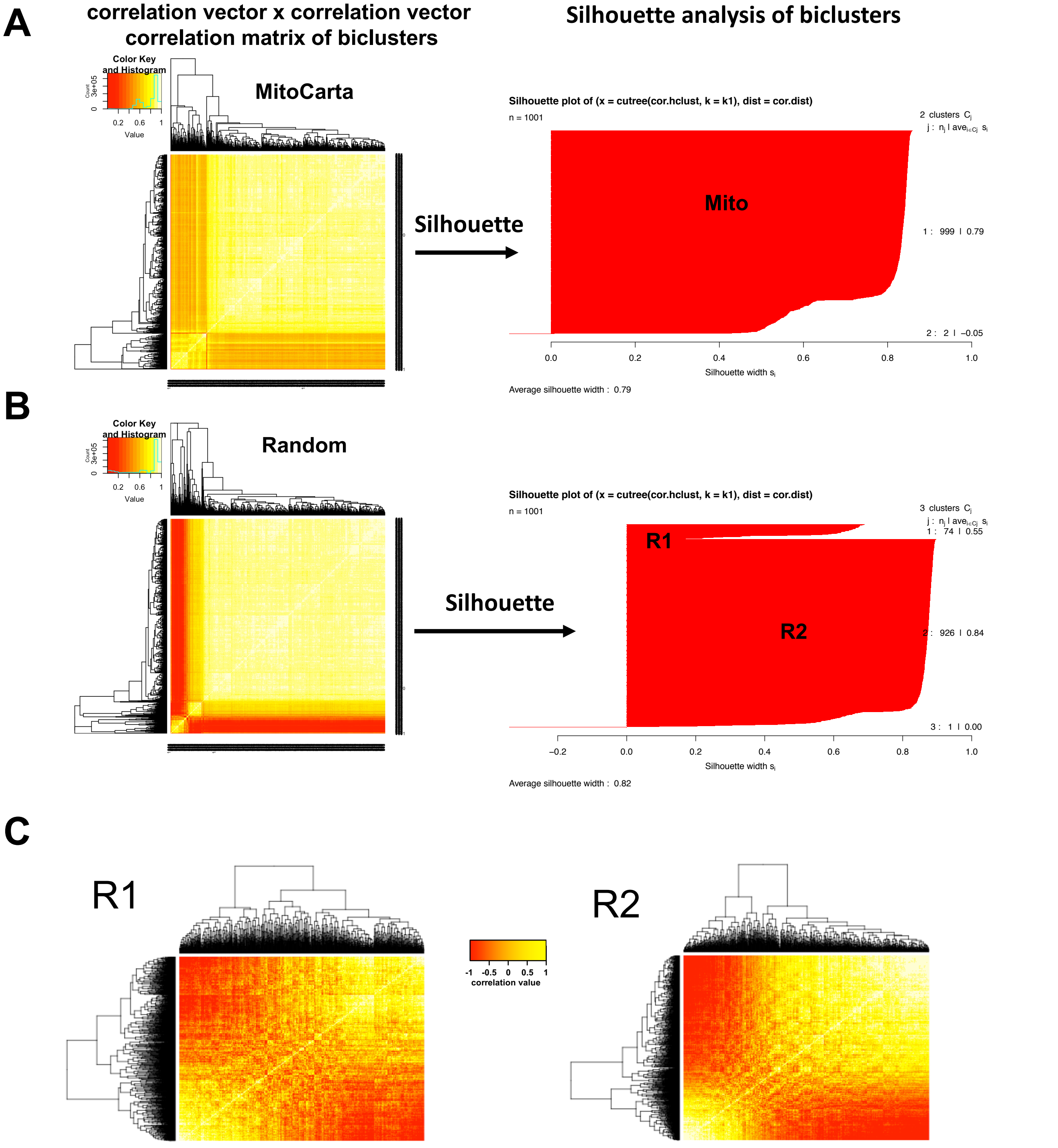

Supplementary Figure S6

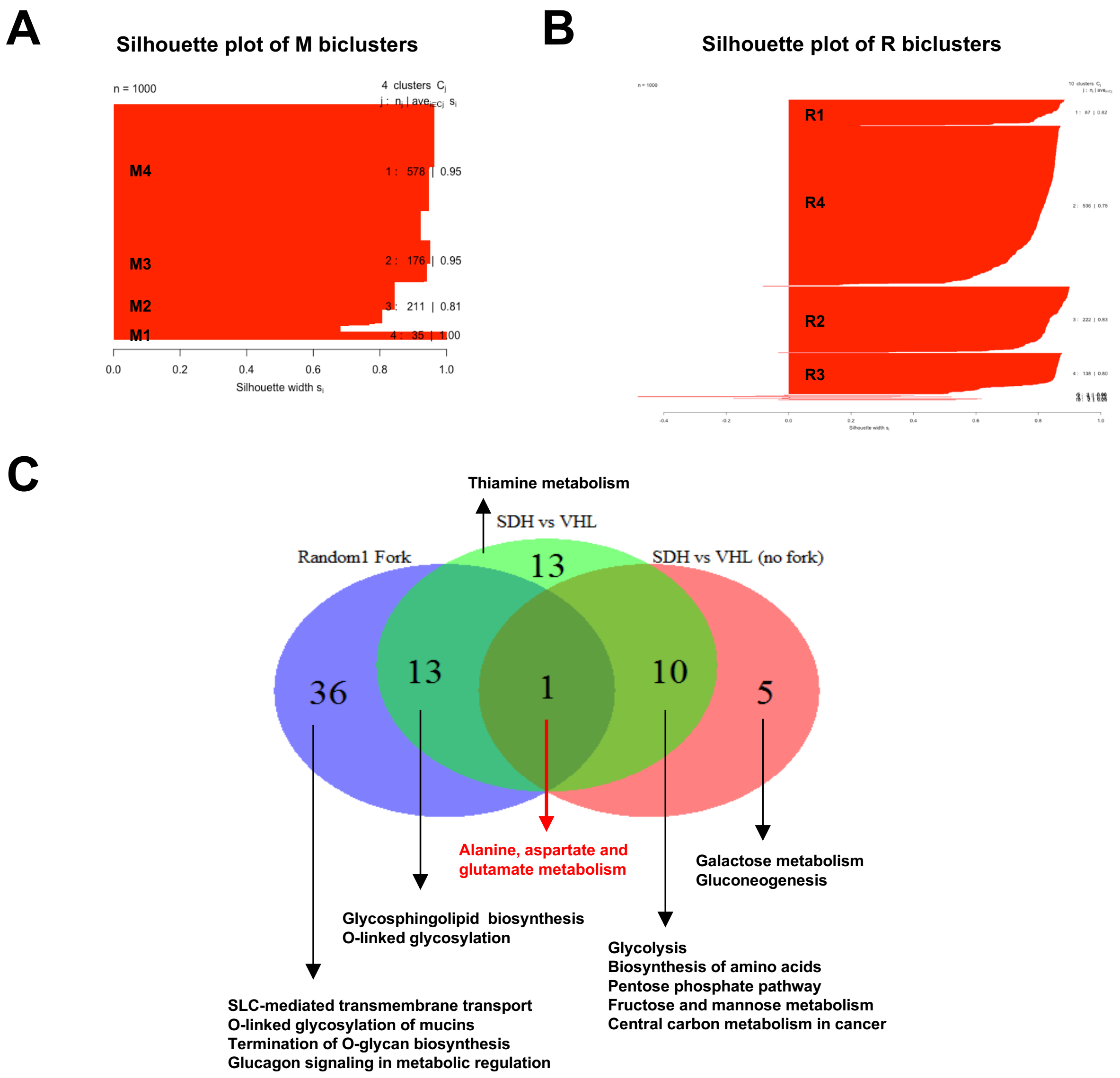

D

			MCbiclust R1	Limma SDH - VHL logFC
GOT1	aspartate aminotransferase, cytoplasmic	EC:2.6.1.1	0.96	1.25
GOT2	aspartate aminotransferase, mitochondrial	EC:2.6.1.1	0.92	0.47
IDH3A	isocitrate dehydrogenase 3 (NAD(+)) alpha	EC:1.1.1.41	0.88	0.37
ACO2	aconitase 2	EC:4.2.1.3	0.86	0.13
CS	citrate synthase	EC:2.3.3.1	0.85	0.59
MDH1	malate dehydrogenase 1	EC:1.1.1.37	0.85	0.32
DLAT	dihydrolipoamide S-acetyltransferase	EC:2.3.1.12	0.84	0.32
PC	pyruvate carboxylase	EC:6.4.1.1	0.80	0.20
PDHA1	pyruvate dehydrogenase (lipoamide) alpha 1	EC:1.2.4.1	0.77	0.27
OGDHL	oxoglutarate dehydrogenase-like	EC:1.2.4.2	0.75	0.17
SDHD	succinate dehydrogenase complex subunit D		0.73	0.10
MDH2	malate dehydrogenase 2	EC:1.1.1.37	0.73	0.25
PCK2	phosphoenolpyruvate carboxykinase 2, mitochondrial	EC:4.1.1.32	0.69	0.16
SUCLG1	succinate-CoA ligase alpha subunit	EC:6.2.1.5 6.2.1.4	0.69	
PDHB	pyruvate dehydrogenase (lipoamide) beta	EC:1.2.4.1	0.64	0.33
PDHA2	pyruvate dehydrogenase (lipoamide) alpha 2	EC:1.2.4.1	0.60	0.14
ACO1	aconitase 1	EC:4.2.1.3	0.50	0.16
SDHC	succinate dehydrogenase complex subunit C		0.30	0.15
DLST	dihydrolipoamide S-succinyltransferase	EC:2.3.1.61	0.30	-0.05
ACLY	ATP citrate lyase	EC:2.3.3.8	0.30	0.03
SDHA	succinate dehydrogenase flavoprotein subunit A	EC:1.3.5.1	0.29	-0.02
FH	fumarate hydratase	EC:4.2.1.2	0.20	0.22
DLD	dihydrolipoamide dehydrogenase	EC:1.8.1.4	0.02	0.10
SUCLA2	succinate-CoA ligase ADP-forming beta subunit	EC:6.2.1.5 6.2.1.4	0.00	-0.12
IDH3G	isocitrate dehydrogenase 3 (NAD(+)) gamma	EC:1.1.1.41	-0.11	0.02
IDH1	isocitrate dehydrogenase (NADP(+)) 1, cytosolic	EC:1.1.1.42	-0.11	-0.07
IDH3B	isocitrate dehydrogenase 3 (NAD(+)) beta	EC:1.1.1.41	-0.31	-0.54
IDH2	isocitrate dehydrogenase (NADP(+)) 2, mitochondrial	EC:1.1.1.42	-0.50	
PCK1	phosphoenolpyruvate carboxykinase 1	EC:4.1.1.32	-0.54	-0.38
SDHB	succinate dehydrogenase complex iron sulfur subunit B	EC:1.3.5.1	-0.54	-0.49
OGDH	oxoglutarate dehydrogenase	EC:1.2.4.2	-0.69	-0.51
SUCLG2	succinate-CoA ligase GDP-forming beta subunit	EC:6.2.1.5 6.2.1.4	-0.80	

SDHx
VHL

Supplementary Figure S7

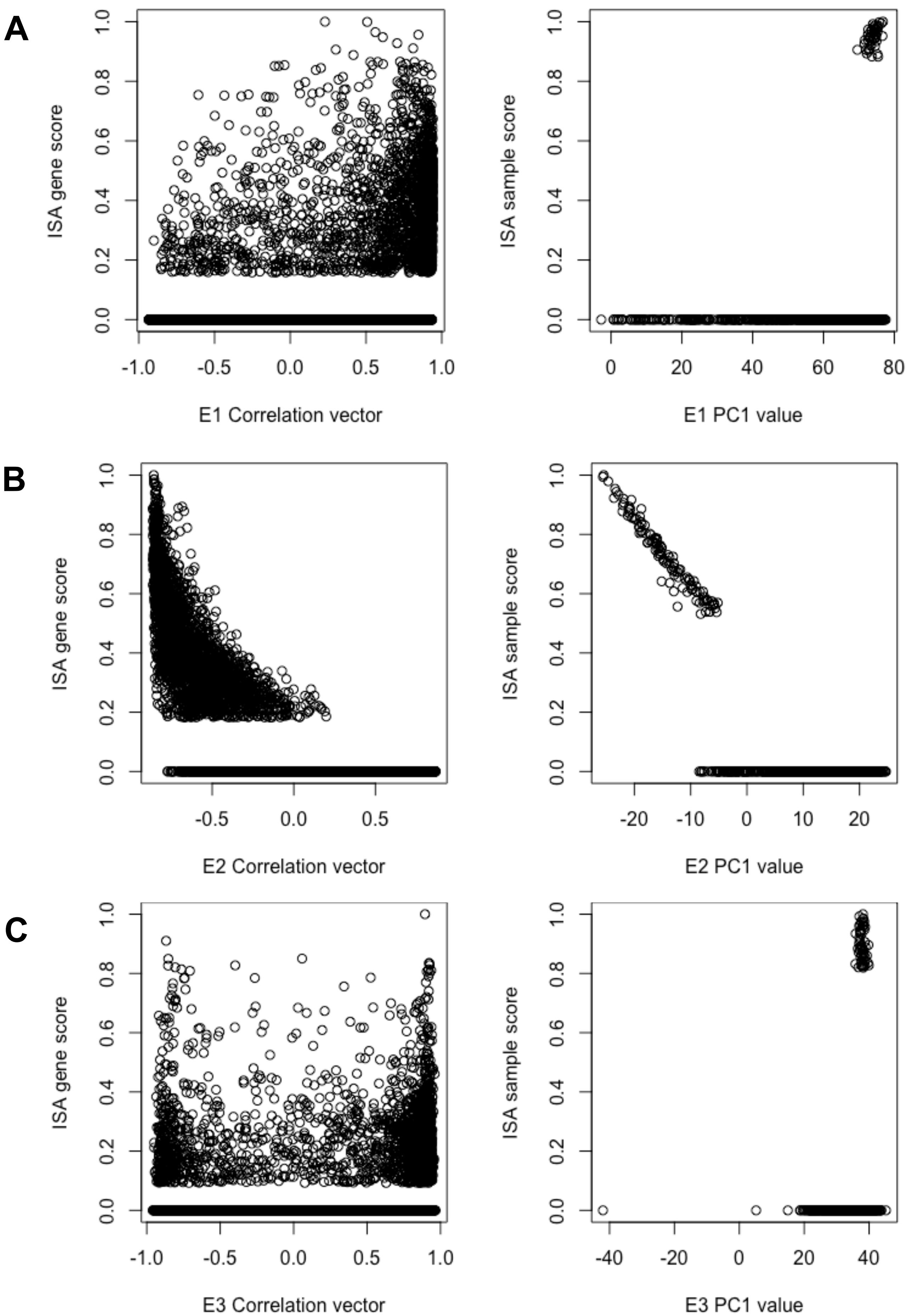

Supplementary Figure S8
